# Influence of Fermentation Methods on Jujube Wine Quality: A Volatile Metabolomics-Based Investigation

**DOI:** 10.3390/foods15203604

**Published:** 2026-10-10

**Authors:** Binbin Li, Qian Mu, Huijing Guo, Wenting Jia, Ya Liu, Xinwen Jin

**Affiliations:** 1 Institute of Agricultural Products Processing, Xinjiang Academy of Agricultural Reclamation Sciences, Shihezi 832000, China; binbinliwork@163.com (B.L.); muxi0199@163.com (Q.M.); 13369932053@163.com (H.G.); 17899316362@163.com (W.J.); 2 School of Food Science, Shihezi University, Shihezi 832003, China

**Keywords:** jujube wine, mixed fermentation, non-*Saccharomyces* yeasts, volatile compounds, GC–MS

## Abstract

Co-fermentation with *Saccharomyces cerevisiae* and selected non-*Saccharomyces* yeasts is an effective approach for producing fruit wines with distinctive aromas. This study aimed to improve the aroma complexity of jujube wine by evaluating multi-strain sequential inoculation strategies. Two indigenous non-*Saccharomyces* strains, *Hanseniaspora uvarum* TD74 and *Metschnikowia pulcherrima* HJ79, were sequentially inoculated for fermentation in combination with *S. cerevisiae*. The physicochemical properties and volatile compound profiles of jujube wines produced under different inoculation conditions were analyzed using HS-SPME-GC–MS, coupled with electronic nose, electronic tongue, and chemometric analyses. Compared with single-strain fermentation, sequential inoculation with the two non-*Saccharomyces* yeasts produced significantly more distinct volatile profiles. In particular, the triple-strain sequential inoculation group (MHS) exhibited higher semi-quantitative levels of most ethyl esters, with a more pronounced fruity aroma in the wine, consistent with the results of sensory analysis. These findings demonstrated that the use of multiple non-*Saccharomyces* yeasts is an effective strategy for enhancing the aromatic diversity and overall sensory quality of jujube wine, offering a theoretical basis for the rational design of optimized starter cultures for industrial jujube wine production.

## 1. Introduction

The aromatic profile of fruit wine is determined by the combined contributions of varietal, fermentative, and aging-derived aromas. Among these, fermentative aroma primarily arises from secondary metabolites produced during yeast metabolism [1]. Although spontaneous fermentation can confer product-specific aroma profiles associated with indigenous microbial communities and local raw materials and enhance wine complexity, its inherent uncontrollability, susceptibility to sluggish fermentation, and prolonged time requirements limit its application in industrial production [2]. Consequently, commercial fruit wine production predominantly relies on pure-culture fermentation with *Saccharomyces cerevisiae* strains. This approach provides a controllable fermentation process and enables efficient sugar-to-ethanol conversion. However, fermentation with a single yeast strain usually results in a relatively defined strain-dependent metabolic profile. Although some yeast strains possess strong aroma-producing capabilities, co-fermentation with multiple yeast species can integrate complementary metabolic pathways and generate a broader range of unique flavor combinations, thereby enhancing aroma complexity and product-specific characteristics [3]. In order to leverage the microbial diversity associated with spontaneous fermentation while also maintaining process controllability, mixed-culture fermentation with indigenous non-*Saccharomyces* yeasts and *S. cerevisiae* has emerged as an effective strategy for improving the flavor quality of fruit wines [4,5]. Non-*Saccharomyces* yeasts enhance wine aroma mainly through their enzymatic activities. They release β-glucosidase, which hydrolyzes non-volatile glycosidic aroma precursors found in fruit, thereby releasing volatile aglycones that add floral and fruity notes to the fermented product [6]. In addition, these yeasts also synthesize esterases that catalyze both the formation and hydrolysis of short-, medium-, and long-chain fatty acid esters, thereby directly shaping the ester composition of fruit wines [7]. The beneficial roles of several non-*Saccharomyces* strains have been well documented. For example, *Torulaspora delbrueckii*, *Hanseniaspora vineae*, and *Lachancea thermotolerans* have been reported to lend fruity and flowery aromas by influencing ester biosynthesis [8]. Similarly, the fermentation of Gewürztraminer wine with β-glucosidase-producing *Candida glabrata* significantly increases its floral and fruity characteristics [9]. Likewise, high enzyme-producing strains such as *Zygosaccharomyces rouxii*, *Meyerozyma guilliermondii*, and *Pichia kudriavzevii* have been shown to increase the contents of ethyl esters, acetate esters, and higher alcohols, thereby intensifying the floral and tropical fruit aromas of kiwifruit wine [10]. Despite their outstanding enzymatic activities and aroma-producing capabilities, most non-*Saccharomyces* yeasts are generally unable to independently accomplish sugar-to-ethanol conversion because of their weak tolerance to winemaking stresses, particularly high sugar and ethanol levels [9]. Consequently, *S. cerevisiae*, with its powerful fermentative capacity and robust stress tolerance, remains the dominant species for industrial alcoholic fermentation. However, the β-glucosidase activity of *S. cerevisiae* is generally lower than that of many non-*Saccharomyces* yeasts [11]. This gap has led to the adoption of sequential inoculation strategies, in which non-*Saccharomyces* yeasts are inoculated during the early stages of fermentation to maximize their enzymatic actions, followed by the addition of *S. cerevisiae* to guarantee complete sugar utilization and efficient ethanol production.

Jujube (*Ziziphus jujuba* Mill.) is an economically important fruit crop cultivated worldwide, with considerable industrial value in China. The fruit has a long history of utilization and is widely consumed as fresh fruit and dried products. Owing to its abundant nutritional and bioactive components, including carbohydrates, vitamins, organic acids, flavonoids, phenolic compounds, and polysaccharides, jujube has attracted increasing attention as a valuable resource for functional foods and value-added products [12]. Recent metabolomic studies have further revealed significant variations in nutritional metabolites among jujube cultivars and production regions, highlighting the potential for quality improvement and diversified utilization of jujube resources [13]. Among its numerous cultivars, “Huizao” is one of the predominant commercial varieties cultivated in Xinjiang, China, owing to its high yield, large fruit size, and desirable quality characteristics [14]. Currently, Huizao is mainly utilized for fresh consumption and dried fruit production, whereas the development of diversified and high-value-added products, particularly fermented beverages, remains relatively limited. Therefore, exploring appropriate processing technologies for Huizao is of great significance for improving resource utilization efficiency, extending the industrial value chain, and enhancing the economic benefits of the jujube industry. The production of fermented jujube wine represents a promising opportunity to increase the added value of Huizao fruit and prolong its shelf life through bioprocessing. As consumer demand for fruit wines continues to grow, flavor innovation has become a key priority for industrial growth. In our previous study, two non-*Saccharomyces* yeast strains with high β-glucosidase activity, *Hanseniaspora uvarum* TD74 and *Metschnikowia pulcherrima* HJ79, were isolated from spontaneously fermenting jujube juice. Their enological characteristics and enzymatic properties were systematically evaluated, demonstrating favorable fermentation adaptability and stable enzymatic activity. The findings highlight their potential as starter cultures for jujube wine production. However, the aroma-enhancing effects of these two strains during jujube wine fermentation and their interactions with *S. cerevisiae* still remain unclear.

Accordingly, the present study employed headspace solid-phase microextraction coupled with gas chromatography–mass spectrometry (HS-SPME-GC–MS), as well as electronic nose, electronic tongue, and chemometric analyses, to systematically evaluate the fermentation kinetics, physicochemical characteristics, and volatile compound composition of jujube wines produced using different yeast combinations. The objectives were to compare the volatile compound profiles at the fermentation endpoint among different inoculation regimes, to clarify the aroma-enhancing effects of non-*Saccharomyces* yeasts during mixed fermentation, and to identify the best-performing inoculation strategy among those tested under the specific laboratory conditions used here, thereby providing a theoretical basis for evaluating mixed-culture starter designs for jujube wine. The findings could potentially support the advancement of novel winemaking techniques.

## 2. Materials and Methods

### 2.1. Yeast Strains and Juice Preparation

Two non-*Saccharomyces* yeast strains previously isolated from Xinjiang jujube, *Metschnikowia pulcherrima* HJ79 and *Hanseniaspora uvarum* TD74, were used in this study. Both exhibited good β-glucosidase activity and favorable enological characteristics. These strains were preserved at the Institute of Agro-Products Processing, Xinjiang Academy of Agricultural Reclamation Sciences (Shihezi, Xinjiang, China). *Saccharomyces cerevisiae* MST, obtained from Angel Yeast Co., Ltd. (Yichang, Hubei, China), served as the reference strain.

Jujube fruits (*Ziziphus jujuba* Mill. cv. Huizao) were harvested from an orchard in Ruoqiang County, Xinjiang, China (39°02′ N, 88°10′ E). Dried jujube fruits (moisture 18.25% ± 1.05%, total soluble solids 65.76% ± 2.34%) were mixed with deionized water at a fruit-to-water ratio of 1:3 (*w*/*v*), soaked for 2 h, and homogenized. Pectinase was added at 0.002% (*w*/*v*), and enzymatic hydrolysis was performed at 45 °C for 2 h. After filtration through a double layer of cheesecloth, the clarified juice had an initial soluble solids content of approximately 17 °Brix, which was then raised to 22 °Brix by adding sucrose. The pH was adjusted to 4.0 using citric acid. The prepared juice was heat-pasteurized at 85 °C for 15 min to control native microorganisms, cooled to room temperature, and stored at −20 °C until subsequent fermentation experiments.

### 2.2. Mixed Fermentation

Seven experimental groups were established: (1) unfermented jujube juice (CK); (2) pure-culture fermentation with *Saccharomyces cerevisiae* MST (Sc, control); (3) pure-culture fermentation with *Metschnikowia pulcherrima* HJ79 (Mp); (4) pure-culture fermentation with *Hanseniaspora uvarum* TD74 (Hu); (5) sequential inoculation of *M. pulcherrima* HJ79 followed by *S. cerevisiae* after 2 days (MS); (6) sequential inoculation of *H. uvarum* TD74 followed by *S. cerevisiae* after 2 days (HS); and (7) simultaneous inoculation of *M. pulcherrima* HJ79 and *H. uvarum* TD74, followed by *S. cerevisiae* after 2 days (MHS). Yeast cultures were prepared at a concentration of 1 × 10^7^ CFU/mL. For single-strain controls (Sc, Mp, Hu), each strain was inoculated at 3% (*v*/*v*). For dual-strain sequential fermentations (HS, MS), the non-*Saccharomyces* strain was first inoculated at 3% (*v*/*v*), followed by *S. cerevisiae* at 3% (*v*/*v*) after 2 days. For the triple-strain fermentation (MHS), *H. uvarum* TD74 and *M. pulcherrima* HJ79 were co-inoculated simultaneously at 1.5% (*v*/*v*) each, giving a total non-*Saccharomyces* inoculum of 3% (*v*/*v*), followed by *S. cerevisiae* at 3% (*v*/*v*) after 2 days. Accordingly, the initial concentration of each strain was 3 × 10^5^ CFU/mL in the single- and dual-strain groups, and 1.5 × 10^5^ CFU/mL for each non-*Saccharomyces* strain (Hu and Mp) and 3 × 10^5^ CFU/mL for *S. cerevisiae* in the MHS group. The “1:1 ratio” refers to the volume ratio of the total initial non-*Saccharomyces* inoculum to the subsequent *S. cerevisiae* inoculum.

Fermentation was conducted in 1-L Erlenmeyer flasks containing 800 mL of jujube juice at 20 °C. The flasks were sealed with air-permeable sealing film. Samples were collected daily throughout fermentation. Total soluble solids were monitored using a handheld refractometer (PAL-1), and the reducing sugar content was determined simultaneously. Fermentation was considered to have reached the endpoint when the residual sugar content remained constant for 72 h. All physicochemical and sensory analyses were performed on clarified and stabilized wines collected at this endpoint.

### 2.3. Physicochemical Parameter Analysis

The pH of the jujube wines was measured using a pH meter (PHS-3C, INESA Scientific Instrument Co., Ltd., Shanghai, China). The levels of reducing sugar and total acidity were analyzed in accordance with the Chinese National Standard GB/T 15038—2006 [15], while the ethanol concentration was quantified following GB 5009.225—2023 [16]. Total phenolic content and total flavonoid content were determined using the Folin–Ciocalteu method and the NaNO_2_-Al(NO_3_)_3_-NaOH colorimetric method, respectively [17,18]. Antioxidant capacity was evaluated by measuring DPPH radical scavenging activity, ABTS^+^ radical scavenging activity, hydroxyl radical scavenging activity, and total reducing power [19]. Color parameters were determined using a colorimeter (WSC-2S, INESA Physical Optics Instrument Co., Ltd., Shanghai, China). The CIE Lab^*^ color parameters, including lightness (L^*^), redness/greenness (a^*^), and yellowness/blueness (b^*^), were recorded. The total color difference (ΔE^*^) between each fermented sample and the unfermented control (CK) was calculated directly from the corresponding L^*^, a^*^, and b^*^ values:

$${\Delta E}^{*}=\sqrt{\left[{(L}_{i}^{*}-{L_{\mathrm{CK}}^{*})}^{2}+(a_{i}^{*}-{a_{\mathrm{CK}}^{*})}^{2}+{(b}_{i}^{*}-{b_{\mathrm{CK}}^{*})}^{2}\right]}$$

where L^*^_i_, a^*^_i_, and b^*^_i_ represent the corresponding color parameters of each fermented wine sample, and L^*^_CK_, a^*^_CK_, and b^*^_CK_ represent those of the unfermented jujube juice control.

### 2.4. Evaluation of Inoculation Strategies

During fermentation, samples were collected every 24 h, and changes in yeast biomass were monitored using the plate count method. Briefly, 1 mL of fermentation broth was serially diluted 10-fold with sterile 0.85% (*w*/*v*) saline, spread onto WLN agar plates, and incubated at 28 °C for 48 h before colony enumeration. Prior to the fermentation experiments, the identities of the three starter strains were confirmed by sequencing of the internal transcribed spacer (ITS) region using primers ITS1/ITS4 and the D1/D2 domain of the 26S rRNA gene, followed by sequence comparison against the NCBI database using the online BLAST service (https://blast.ncbi.nlm.nih.gov/Blast.cgi, accessed on 7 October 2026). Pure cultures of the three strains were also independently grown on WLN agar under the same incubation conditions to establish reference colony morphologies. Under these conditions, *S. cerevisiae* MST produced creamy-white, smooth, and raised colonies, *H. uvarum* TD74 produced bright-green colonies, whereas *M. pulcherrima* HJ79 produced characteristic red-pigmented colonies associated with pulcherrimin formation. These visually distinct phenotypes were used as reference characteristics for population enumeration in mixed fermentations. Specifically, colonies recovered from mixed-culture samples were presumptively assigned to *S. cerevisiae*, *H. uvarum*, or *M. pulcherrima* by comparison with the corresponding pure-culture controls cultured in parallel under identical conditions.

β-Glucosidase activity was determined according to the method reported by Hu et al. [20]. Briefly, 5 mL of fermentation broth was centrifuged at 12,000 r/min and 4 °C for 15 min, and the supernatant was collected as the crude enzyme extract. The reaction mixture contained 200 μL of crude enzyme extract and 250 μL of citrate–phosphate buffer (pH 5.0) containing p-nitrophenyl-β-D-glucopyranoside (PNPG) at a final concentration of 1 mmol/L. After incubation at 50 °C for 10 min, the reaction was terminated by adding 1 mL of 1 mol/L Na_2_CO_3_. The released p-nitrophenol (PNP) was quantified spectrophotometrically at 400 nm against a blank prepared with heat-inactivated crude enzyme extract. PNP concentrations were calculated from a calibration curve (A_400_ = 0.01246·C_PNP_ + 0.00486, R^2^ = 0.9998), which was constructed using serial PNP standards (10–70 µM) prepared in 1 mol/L Na_2_CO_3_. One unit (U) of β-glucosidase activity was defined as the amount of enzyme required to release 1 μmol of PNP per minute under the assay conditions [20].

### 2.5. HS-SPME-GC-MS Analysis

Volatile compounds were extracted by HS-SPME and analyzed using a GC–MS system (GCMS-QP2020 NX, Shimadzu, Japan) equipped with a DB-WAX capillary column (30 m × 0.25 mm × 0.25 μm, Agilent Technologies, Santa Clara, CA, USA). Briefly, 3 mL of sample, 0.6 g of NaCl, and 5 μL of 4-octanol (0.45 mg/mL, internal standard) were added to a headspace vial. The mixture was equilibrated at 45 °C under continuous stirring for 15 min, after which a preconditioned 65-μm PDMS/DVB fiber was exposed to the headspace for extraction at 45 °C for 45 min. The analytes were subsequently thermally desorbed in the GC injection port for 5 min. The GC–MS operating conditions were set as previously described [21]. Volatile compounds were identified by mass spectral library matching (NIST 8.0 and Wiley 7) and retention index (RI) comparison [22]. Semi-quantification was performed using the peak area ratio relative to the internal standard (4-octanol). Because absolute quantification was not performed, absolute OAVs were not calculated. Instead, relative odor activity values (ROAVs) were calculated based on semi-quantitative levels and literature odor thresholds:

$${ROAV}_{i}=\frac{{OAV}_{i}}{{OAV}_{max}}\times 100$$

where OAV*_i_* = C*_i_*/T*_i_*, C*i* is the semi-quantitative level, and T*i* is the odor threshold from the literature. ROAV is a relative indicator of odor contribution within each sample.

### 2.6. Electronic Nose (E-Nose) and Electronic Tongue (E-Tongue) Analyses

The aroma profile of the jujube wines was evaluated using an E-Nose (CNose, Shanghai Bosin Tech, Shanghai, China) equipped with 28 sensors. The sensor array and its corresponding target compounds are provided in Supplementary Table S1. The analysis was performed according to a previously reported method [23]. Briefly, 10 mL of sample was transferred into a headspace vial, sealed, and equilibrated at room temperature for 30 min. Clean air was employed as the carrier gas at a flow rate of 1.0 L/min; the sample analysis time and sensor cleaning time were both set to 120 s; and the sensor cleaning flow rate was maintained at 6 L/min.

Taste characteristics were evaluated using an E-Tongue (Ctongue, Shanghai Bosin Tech, Shanghai, China) equipped with six inert-metal working electrodes (Pt, Au, Pd, Ti, W, and Ag), a platinum auxiliary electrode, and a Ag/AgCl reference electrode. Based on a previously reported method, with minor modifications [24], 15 mL of jujube wine was transferred into a 50-mL beaker, and the sensor array was immersed directly into the test solution. The data acquisition parameters were as follows: sensor amplification factor, 100×; measurement time, 138 s; and three replicate measurements for each sample. The sensors were rinsed with ultrapure water for 15 s between successive measurements. The response values of each sensor were recorded automatically by the instrument software. Each electrode generated a transient potential response under a large-amplitude pulse signal (+1 V to −1 V, 0.2 V per step). From each response curve, three characteristic parameters (maximum potential, steady-state response, and response slope) were extracted, yielding 18 response variables (P1–P18) that served as the input for PCA. P1–P18 are extracted variables from six physical electrodes, not 18 independent sensors.

### 2.7. Sensory Analysis

Following the method reported by Han et al. [25], sensory evaluation was performed by a trained panel of 10 assessors (five men and five women, aged 22–35 years). Prior to the formal evaluation, the panelists—all faculty members or students specializing in oenology—completed three training sessions (each approximately 2 h) to familiarize themselves with the sensory characteristics of jujube wine, including the 10 attribute definitions and reference standards listed in Table S2. All wine samples were equilibrated at room temperature for 1 h. All seven samples were evaluated in a single session; 20 mL of each sample was poured into standard wine-tasting glasses, randomly coded, and presented to the panelists. The serving order was randomized independently for each panelist to minimize order and carryover effects. Sensory attributes were evaluated using a 10-point scale (0 = not perceptible, 9 = extremely high). Through three consensus discussions, the panel established standardized definitions and reference standards for 10 sensory attributes (Table S2), which were classified into two categories: aroma and taste. During the evaluation, assessors rinsed their mouths with water and rested for 10 min between samples. The analysis was conducted in accordance with accepted ethical standards for sensory evaluation. Participation was voluntary, and written informed consent was provided by all participants. All personal information of the participants was anonymized and appropriately protected. Consent for publication of the experimental data was obtained from all participants prior to manuscript submission.

### 2.8. Statistical Analysis

All experiments were performed in triplicate, and the results are expressed as the mean ± standard deviation (SD). Statistical analyses were performed using SPSS 26.0 software (IBM, Armonk, NY, USA). For time-course fermentation data, a two-way repeated-measures ANOVA was performed with inoculation treatment and fermentation time as fixed factors, followed by Duncan’s multiple range test for pairwise comparisons at each time point (*p* < 0.05). For endpoint physicochemical and volatile data, one-way ANOVA followed by Duncan’s test was used. Data visualization was performed using Origin 2024 (OriginLab Corporation, Northampton, MA, USA) and the Metware Cloud Platform.

## 3. Results and Discussion

### 3.1. Yeast Population Dynamics and β-Glucosidase Activity

**Figure 1:**
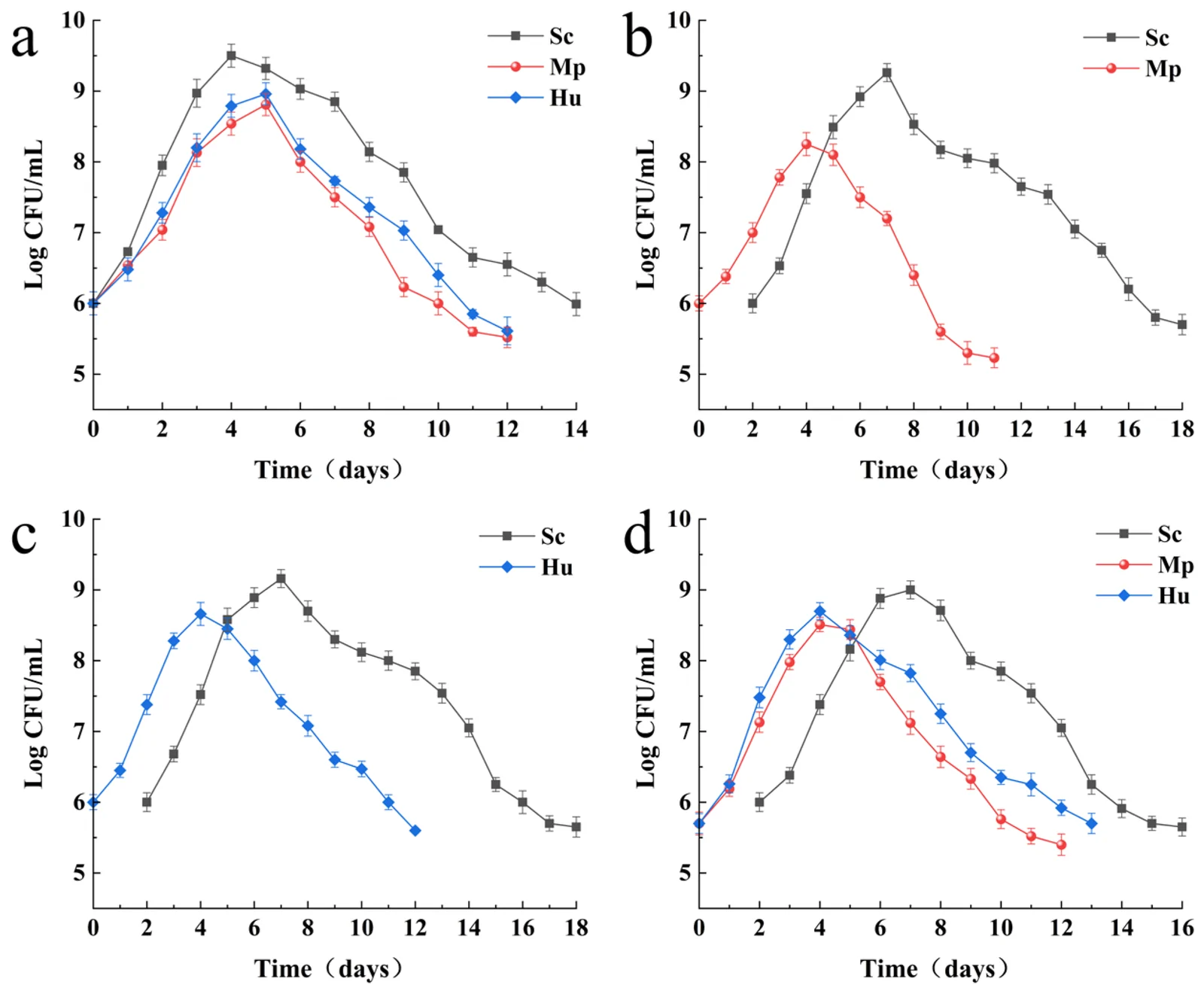
Dynamic changes in yeast biomass under different inoculation conditions. (**a**) Mono-culture fermentation of Sc, Hu, and Mp; (**b**) sequential co-culture fermentation of MS; (**c**) Sequential co-culture fermentation of HS; (**d**) sequential co-culture fermentation of MHS. Data are presented as mean ± SD (*n* = 3). Sc, *S. cerevisiae* MST; Mp, *M. pulcherrima* HJ79; Hu, *H. uvarum* TD74; MS, sequential inoculation of *M. pulcherrima* HJ79 followed by *S. cerevisiae* MST; HS, sequential inoculation of *H. uvarum* TD74 followed by *S. cerevisiae* MST; MHS, simultaneous inoculation of *M. pulcherrima* HJ79 and *H. uvarum* TD74 followed by *S. cerevisiae* MST. Two-way repeated-measures ANOVA showed significant effects of treatment, time, and treatment × time interaction for all four panels (all *p* < 0.001; detailed F values in Table S3).

**Figure 2:**
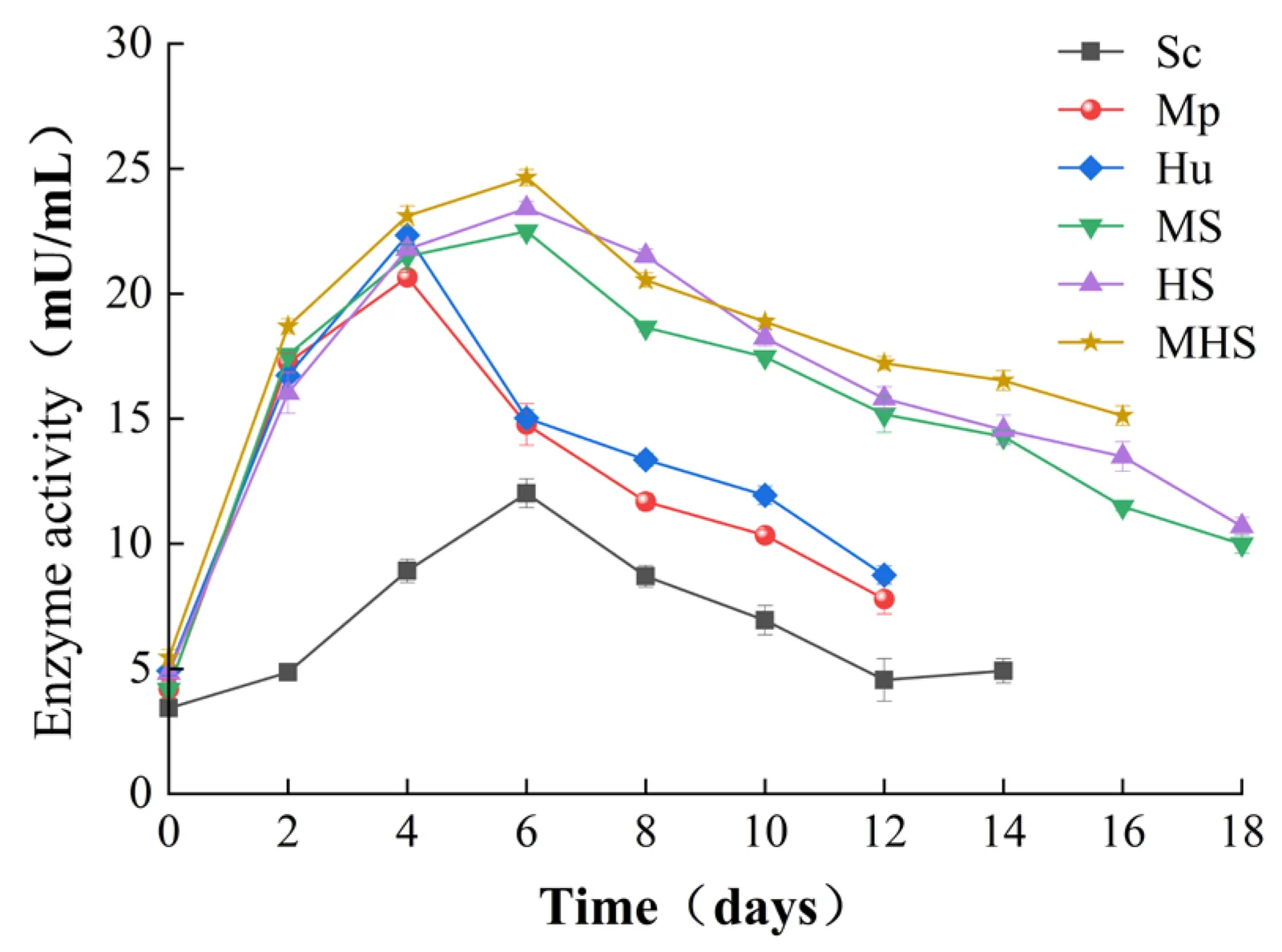
Changes in β-glucosidase activity during fermentation under different inoculation conditions. Data are presented as mean ± SD (*n* = 3). Sc, *S. cerevisiae* MST; Mp, *M. pulcherrima* HJ79; Hu, *H. uvarum* TD74; MS, sequential inoculation of *M. pulcherrima* HJ79 followed by *S. cerevisiae* MST; HS, sequential inoculation of *H. uvarum* TD74 followed by *S. cerevisiae* MST; MHS, simultaneous inoculation of *M. pulcherrima* HJ79 and *H. uvarum* TD74 followed by *S. cerevisiae* MST. Two-way repeated-measures ANOVA showed significant effects of treatment (F(5, 84) = 2216.5, *p* < 0.001), time (F(6, 84) = 2770.2, *p* < 0.001), and treatment × time interaction (F(30, 84) = 115.0, *p* < 0.001).

During single-culture fermentation (Figure 1a), *S. cerevisiae* MST grew rapidly, reaching a peak biomass of 9.5 Log CFU/mL on day 4, followed by a continuous decline. Meanwhile, *H. uvarum* TD74 and *M. pulcherrima* HJ79 grew relatively slowly, attaining peak biomass values on day 5 (8.81 and 8.96 Log CFU/mL, respectively). During dual-strain sequential fermentation, the growth of both non-*Saccharomyces* yeasts was inhibited to a certain degree. The biomass of the non-*Saccharomyces* strains was markedly lower than that of *S. cerevisiae*, exhibiting a declining trend during the later stages of fermentation. However, *M. pulcherrima* HJ79 was more strongly inhibited, with its peak biomass reaching only 8.25 Log CFU/mL (Figure 1b,c). During the triple-strain sequential fermentation (Figure 1d), the biomass of both non-*Saccharomyces* yeasts was higher than that in their respective dual-strain fermentation groups. Moreover, the rate of decline was slower, and the fermentation time was shortened. Concurrently monitored β-glucosidase activity showed trends largely consistent with the yeast population dynamics (Figure 2). Following *S. cerevisiae* inoculation, both β-glucosidase activity and the biomass of non-*Saccharomyces* yeasts decreased rapidly, suggesting that their biomass may be closely associated with β-glucosidase activity.

### 3.2. Changes in Sugar and Ethanol Levels During Fermentation

**Figure 3:**
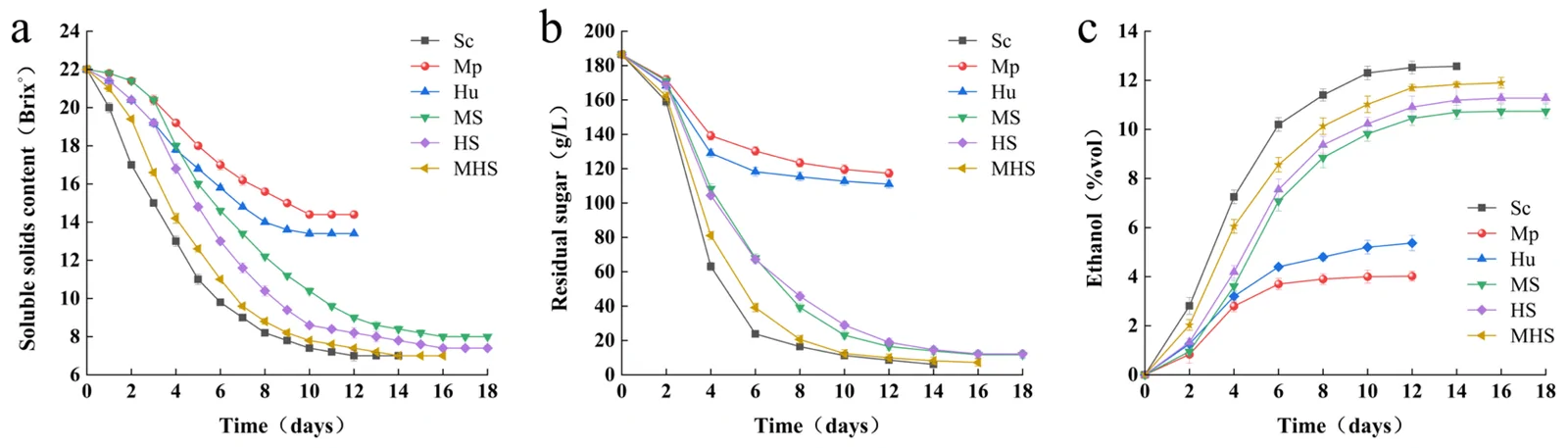
Changes in soluble solids (**a**), reducing sugar (**b**), and alcohol content (**c**) during fermentation under different inoculation conditions. Data are presented as mean ± SD (*n* = 3). Sc, *S. cerevisiae* MST; Mp, *M. pulcherrima* HJ79; Hu, *H. uvarum* TD74; MS, sequential inoculation of *M. pulcherrima* HJ79 followed by *S. cerevisiae* MST; HS, sequential inoculation of *H. uvarum* TD74 followed by *S. cerevisiae* MST; MHS, simultaneous inoculation of *M. pulcherrima* HJ79 and *H. uvarum* TD74 followed by *S. cerevisiae* MST. Two-way repeated-measures ANOVA showed significant effects of treatment, time, and treatment × time interaction for all three parameters (all *p* < 0.001; detailed F values in Table S3).

The dynamic changes in sugar and ethanol concentrations were monitored during single-strain and mixed-culture fermentation. The Sc group exhibited a faster decline in SSC compared with the other fermentation groups, consistent with the strong fermentative capacity of *S. cerevisiae*. From day 0 to day 4, SSC decreased from 22 °Brix to 13 °Brix in the Sc group (Figure 3), indicating active sugar consumption during the early fermentation stage. At the endpoint, the Sc group achieved a residual sugar of 6.1 g/L and an ethanol concentration of 12.57% vol (Table 1). In contrast, the non-*Saccharomyces* single-culture groups (Mp and Hu) showed weak fermentation capacity. After 6 days of fermentation, when ethanol concentration reached approximately 4% vol, SSC reduction slowed markedly, suggesting reduced sugar utilization under increasing ethanol stress. By the endpoint (residual sugar stable for 72 h), both the Mp and Hu groups still retained more than 110 g/L residual sugar, with ethanol below 6% vol. This indicated that fermentation had ceased due to incomplete sugar utilization rather than reaching completion. These findings demonstrated the inability of these strains to independently complete the fermentation of high-sugar substrates. However, sequential inoculation effectively combined the advantages of both yeast types, such that substrate consumption was markedly accelerated after the inoculation of *S. cerevisiae*. Both the HS and MS groups completed fermentation within 18 days. Meanwhile, the MHS group ultimately reached an SSC of 7.0 °Brix, a residual sugar content of 7.2 g/L, and an ethanol concentration of 11.9% vol. These results indicated that *S. cerevisiae* MST may act as the primary sugar consumer during triple fermentation and that non-*Saccharomyces* yeasts may interfere with its sugar consumption efficiency.

### 3.3. Physicochemical Properties of Jujube Wine

Acids are key components that serve as the flavor backbone of fruit wine. Hence, an appropriate level of acidity imparts freshness and complexity to wines. In this study, the total acidity of all fermented samples was higher than that of jujube juice. Notably, total acidity was consistently higher in samples fermented with non-*Saccharomyces* yeasts than in the Sc group samples (4.5 g/L), consistent with their stronger acid-producing characteristics. The pH values in all other fermented groups were lower than the pH value in the Sc group (Table 1). This result is consistent with previous reports that non-*Saccharomyces* yeasts typically produce higher levels of organic acids, particularly acetic acid, compared with pure *S. cerevisiae* cultures [26,27].

**Figure 4:**
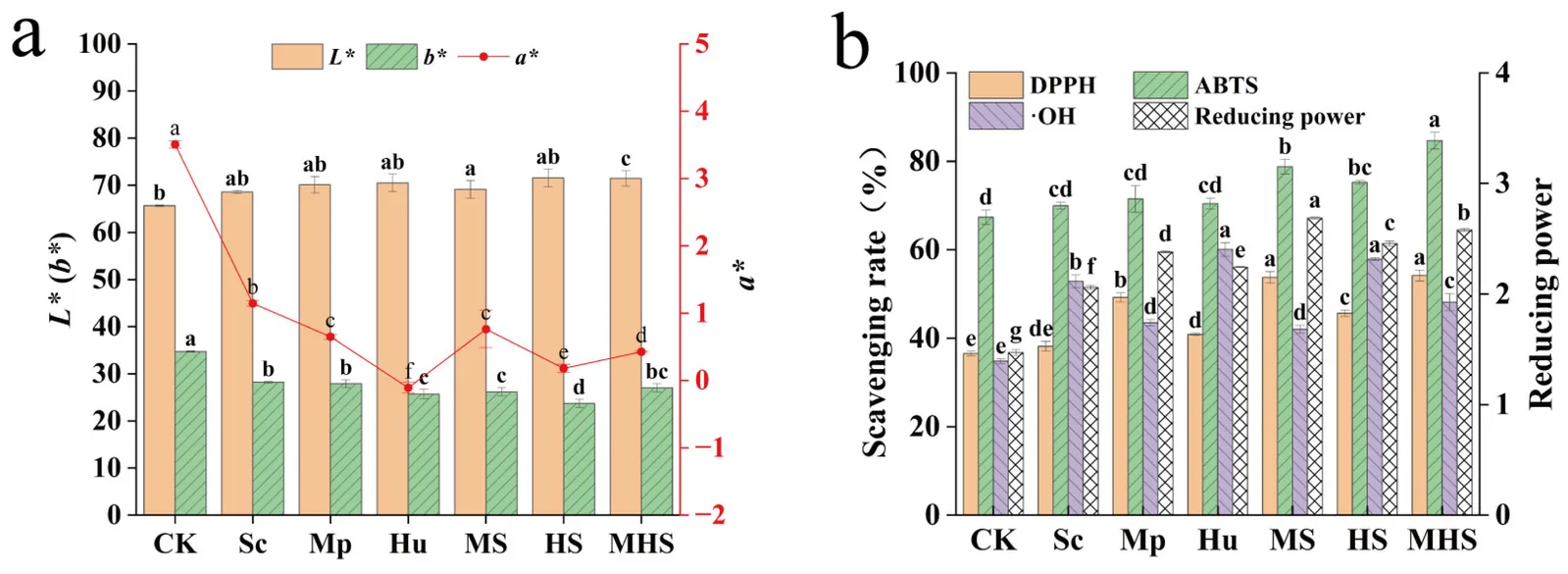
Color parameters and antioxidant activities of jujube wines under different inoculation conditions. (**a**) CIE L^*^a^*^b^*^ color parameters; (**b**) DPPH, ABTS^+^, and hydroxyl radical (•OH) scavenging activities and reducing power. Data are presented as mean ± SD (*n* = 3). Different letters indicate significant differences among treatments (Duncan’s test, *p* < 0.05). CK, unfermented jujube juice; Sc, *S. cerevisiae* MST; Mp, *M. pulcherrima* HJ79; Hu, *H. uvarum* TD74; MS, sequential inoculation of *M. pulcherrima* HJ79 followed by *S. cerevisiae* MST; HS, sequential inoculation of *H. uvarum* TD74 followed by *S. cerevisiae* MST; MHS, simultaneous inoculation of *M. pulcherrima* HJ79 and *H. uvarum* TD74 followed by *S. cerevisiae* MST.

Color is an important factor influencing consumer acceptance for fruit wine. Compared with CK, all fermented samples showed increased L^*^ values, indicating enhanced lightness after fermentation (Figure 4a). Meanwhile, both a* and b* values decreased compared with CK, indicating reduced redness and yellowness (Table 1). The ΔE* values of the fermented wines relative to CK ranged from 7.52 to 12.98 (Table 1). Among all fermentation treatments, the HS group had the highest ΔE^*^ value (12.98), followed by Hu (10.91), MHS (10.15), MS (9.69), Mp (8.67), and Sc (7.52). Since all ΔE^*^ values were greater than 5, the color differences between the fermented wines and the unfermented control were visually distinguishable [28]. These results indicate that different inoculation strategies induced distinct changes in the color characteristics of jujube wine [29].

Polyphenols can influence the sensory characteristics of fruit wine and provide several health benefits [22]. Hence, their content is an important indicator of fruit wine quality. As shown in Table 1, the total phenolic and total flavonoid contents of all fermentation groups were higher than those of the CK group, indicating that fermentation promoted the dissolution of phenolic compounds. The total phenolic content of the mixed-culture fermentation groups was significantly higher than that of all single-culture groups (*p* < 0.05). Among them, the MHS group exhibited the highest total phenolic content, which was 33.0% higher than that of the CK group. This improvement could be attributed to its higher β-glucosidase activity, leading to the effective hydrolysis of bound polyphenols [30]. Notably, the total flavonoid content of the groups fermented with non-*Saccharomyces* yeasts remained higher than that of the Sc group, indicating that fermentation with these strains was associated with higher measured flavonoid contents.

The antioxidant activity of jujube wine is closely associated with the presence of bioactive substances, including phenolic compounds. These compounds can scavenge free radicals by inhibiting initiation and breaking chain propagation, or suppress free radical formation by chelating metal ions, reducing hydrogen peroxide levels, and quenching superoxide and singlet oxygen [31]. The DPPH, ABTS, and hydroxyl radical (•OH) scavenging activities of jujube wines prepared using different inoculation strategies are shown in Figure 4b. Notably, sequential fermentation led to higher radical scavenging activities than single-culture fermentation, confirming its potential for enhancing the antioxidant capacity of jujube wine. Among the various groups, the MHS group exhibited the highest DPPH and ABTS scavenging activities, likely owing to its higher phenolic content [10]. Interestingly, the •OH scavenging activity showed a slightly different pattern, as it was the highest in the Hu group (60.07%), followed by the HS group (57.85%). Meanwhile, the MS group demonstrated the highest reducing power (2.69).

### 3.4. Effect of Sequential Fermentation on the Volatile Aroma Compounds in Jujube Wine

#### 3.4.1. Composition Analysis

**Figure 5:**
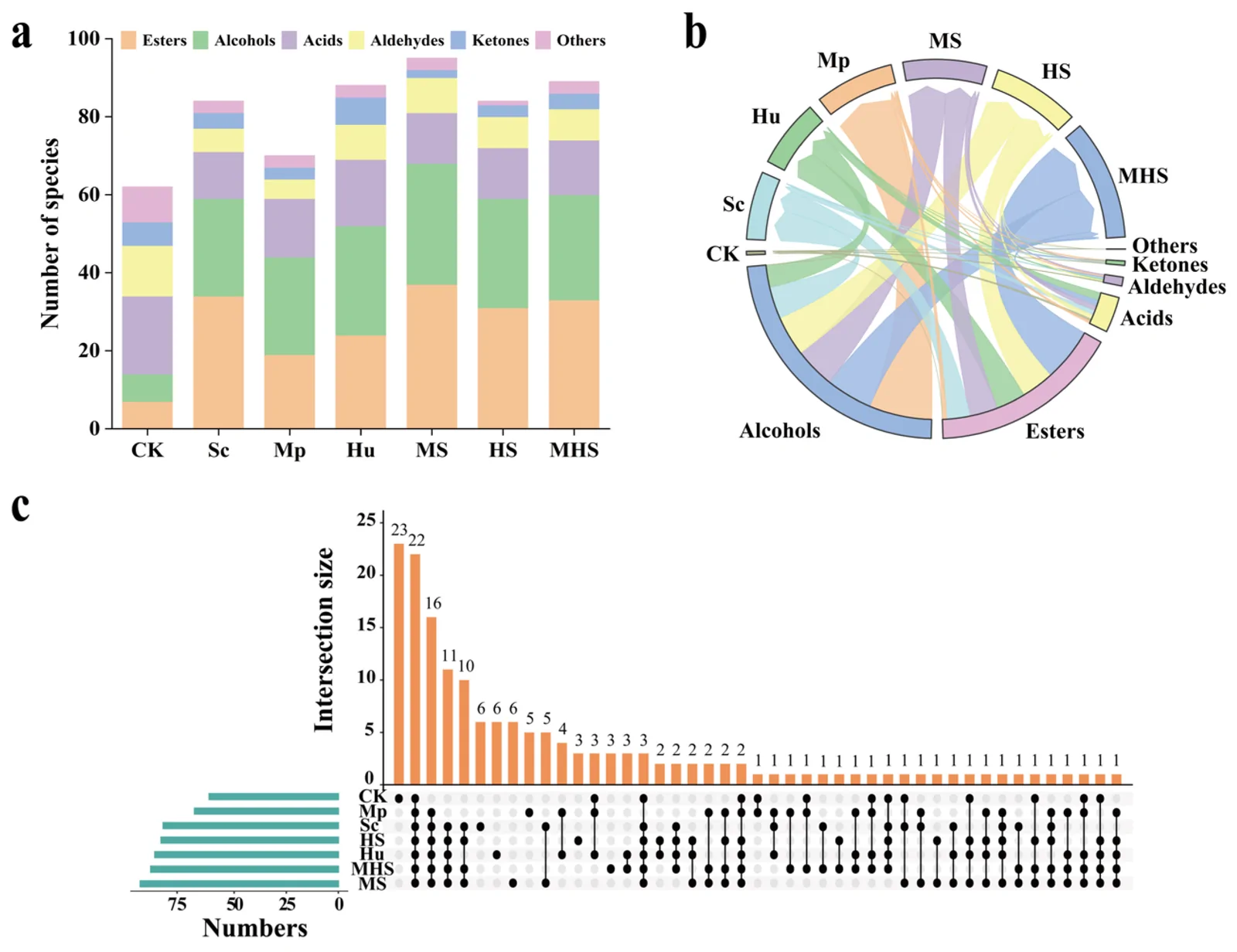
Composition of volatile compounds in different jujube wines. (**a**) Number of volatile compounds belonging to different categories; (**b**) distribution of various types of compounds based on semi-quantitative data; (**c**) number of common and unique compounds. CK, unfermented jujube juice; Sc, *S. cerevisiae* MST; Mp, *M. pulcherrima* HJ79; Hu, *H. uvarum* TD74; MS, sequential inoculation of *M. pulcherrima* HJ79 followed by *S. cerevisiae* MST; HS, sequential inoculation of *H. uvarum* TD74 followed by *S. cerevisiae* MST; MHS, simultaneous inoculation of *M. pulcherrima* HJ79 and *H. uvarum* TD74 followed by *S. cerevisiae* MST.

Volatile compounds are key contributors to the quality of fruit wine, and their composition, concentration, and proportions collectively define the stylistic attributes of the final product [32]. In total, 164 volatile compounds were identified in jujube juice and jujube wine samples, including 51 esters, 43 alcohols, 26 acids, 19 aldehydes, 10 ketones, and 15 other compounds (Table S4 and Figure 5a). As shown in Figure 5b, all fermentation methods significantly increased the total semi-quantitative level of volatile compounds compared with jujube juice. Moreover, the mixed-culture groups had significantly higher total semi-quantitative levels of volatile compounds than the Sc single-culture group. Notably, the MHS group exhibited the highest total semi-quantitative level of volatile compounds (6700.65 μg/L), while the MS group contained the richest variety, with 95 compounds detected in these samples. In total, 22 compounds were common to all groups and were identified as the basic aroma components of jujube (Figure 5c). Among them, isoamyl alcohol showed the highest semi-quantitative level (CK group: 0.69 μg/L; fermented groups: 997.58–1931.93 μg/L) (Table S4).

**Figure 6:**
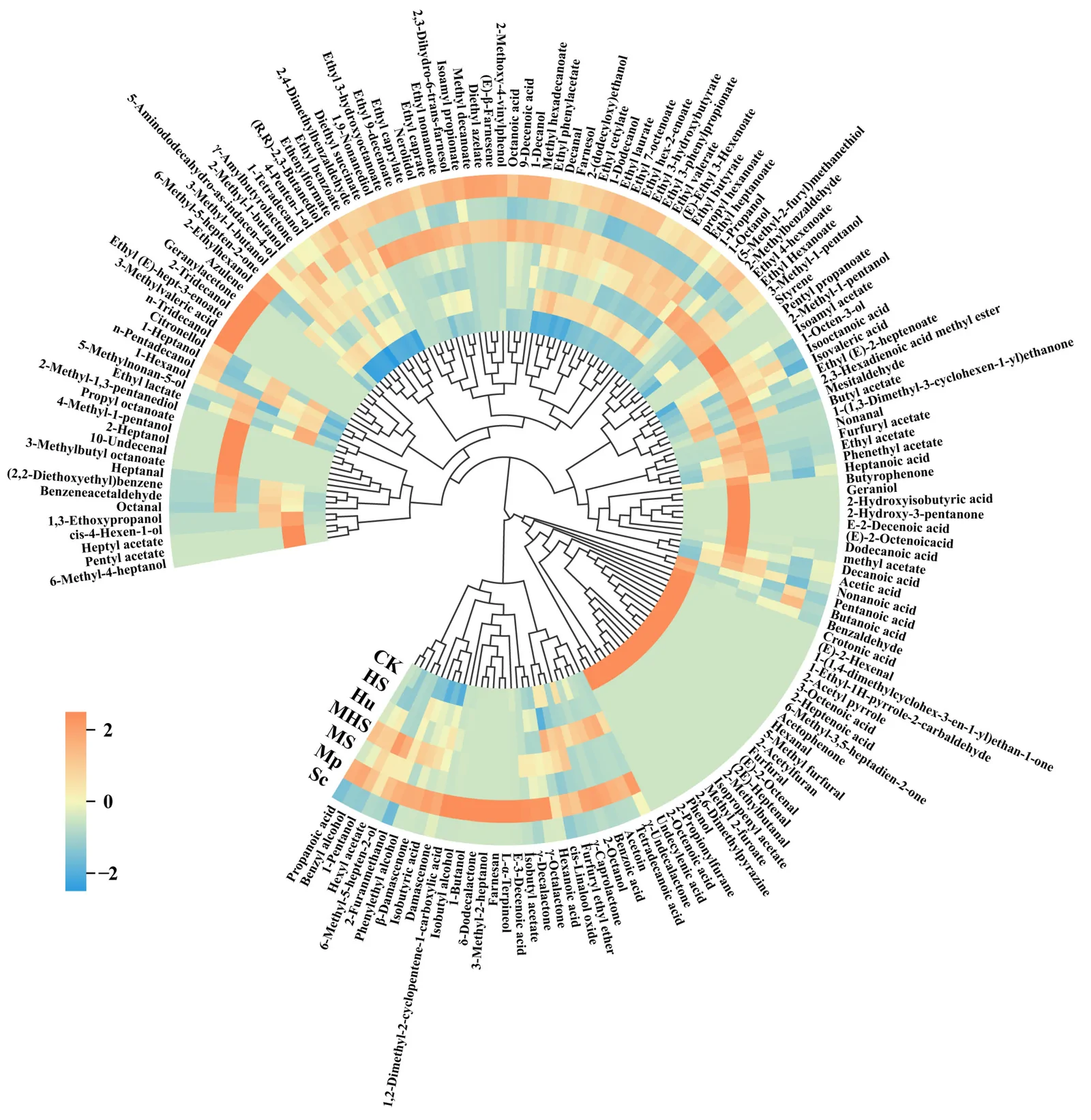
Cluster heatmap of volatile compounds (semi-quantitative levels) found in different jujube wines. CK, unfermented jujube juice; Sc, *S. cerevisiae* MST; Mp, *M. pulcherrima* HJ79; Hu, *H. uvarum* TD74; MS, sequential inoculation of *M. pulcherrima* HJ79 followed by *S. cerevisiae* MST; HS, sequential inoculation of *H. uvarum* TD74 followed by *S. cerevisiae* MST; MHS, simultaneous inoculation of *M. pulcherrima* HJ79 and *H. uvarum* TD74 followed by *S. cerevisiae* MST.

Esters were the most abundant class of volatile compounds found in jujube wine (Figure 5b). Previous studies have demonstrated that esters improve the aroma of fruit wines, acting as primary contributors of fruity and sweet notes [33]. In this study, 51 esters were identified in jujube wine, of which 44 were derived from the fermentation process and 26 were detected exclusively in the mixed-culture fermentation groups. Ethyl esters, which are primarily produced via the alcohol acyltransferase-catalyzed condensation of acyl-CoA and ethanol, were found to be the predominant esters in jujube wine. The highest semi-quantitative level of ethyl esters was detected in the MHS group (3337.80 μg/L), followed by the HS group (2004.11 μg/L). Ethyl acetate, ethyl octanoate, and ethyl hexanoate were the three predominant ethyl esters, collectively accounting for over half of the total semi-quantitative level of ethyl esters. Ethyl acetate is known to be mainly produced by the enzymatic esterification of acetic acid and ethanol, imparting distinctive apple and pineapple aromas [34]. Compared with the Sc group, all three mixed-culture fermentation groups significantly increased the semi-quantitative levels of ethyl acetate in jujube wine. Notably, the semi-quantitative level of ethyl acetate in MHS samples reached 2035.95 μg/L, likely owing to the higher enzymatic activities of the fermentation strains. Notably, unlike ethyl esters, acetate esters are derived from reactions catalyzed by alcohol-O-acetyltransferases, and their production during fermentation is strongly influenced by acetyltransferase activity, the carbon-to-nitrogen ratio of the medium, and the concentrations of unsaturated fatty acids and free oxygen [35]. As shown in Figure 6, the production of most acetate esters was tightly linked to the action of non-*Saccharomyces* yeasts. Only two acetate esters were present in the CK group, whereas five were detected in the Sc group. The predominant acetate esters in jujube wine were isoamyl acetate, hexyl acetate, and isobutyl acetate, with all three showing substantially elevated semi-quantitative levels after fermentation [36]. However, hexyl acetate—which imparts fruity notes to jujube wine—was only detected after fermentation with *M. pulcherrima*.

Higher alcohols are another major class of volatile compounds found in jujube wine. These compounds primarily influence the overall flavor profile of wine by accentuating ester aromas and imparting a full-bodied mouthfeel [37]. As shown in Figure 6, all fermentation groups showed significantly elevated semi-quantitative levels of higher alcohols. Among them, the mixed-culture fermentation groups exhibited distinct superiority in terms of both the composition and semi-quantitative levels of these compounds. The MHS group contained the highest total semi-quantitative level of higher alcohol content (2891.33 μg/L), whereas the Hu single-culture group had the lowest semi-quantitative level (1385.39 μg/L). 3-Methyl-1-butanol, phenylethyl alcohol, and 2-methylbutanol emerged as the main contributors to the observed differences. These alcohols are known to directly impart rich fruity and floral aromas to wine and can also serve as precursors for esterification reactions.

The volatile acids in fruit wine are primarily derived from the raw material itself or are produced through microbial metabolism. An appropriate level of volatile acids confers a soft and delicate mouthfeel to fruit wine, whereas excessively high levels can lead to pungent and vinegar-like notes [38]. In this study, the Hu group exhibited the highest semi-quantitative level of volatile acids, suggesting that this strain may promote the accumulation of short- and medium-chain fatty acids. Acetic acid, octanoic acid, and decanoic acid were the predominant acids detected in jujube wine. However, due to their high odor thresholds, their contributions to sourness and creamy notes appeared to be relatively limited.

In addition to the abovementioned compounds, ketones and aldehydes are also key components influencing fruit wine flavor, contributing to aromatic complexity, sensory balance, and quality stability. In this study, benzeneacetaldehyde and β-damascenone showed significantly elevated semi-quantitative levels in the MS (314.65 μg/L) and Mp (47.76 μg/L) groups, respectively, imparting honey and rose-like aromas to the jujube wine. However, acetoin was only detected in the Mp and Hu single-culture groups, while styrene was exclusively present in the MHS group. These findings reflected the divergence of metabolic pathways within the medium in the presence of different strain combinations.

#### 3.4.2. ROAV Analysis

To further evaluate the potential contribution of volatile compounds to the aroma characteristics of different jujube wines, relative odor activity values (ROAVs) were calculated based on the semi-quantitative results obtained by GC–MS analysis. Compounds with ROAV ≥ 1 were considered as potential aroma-active compounds [39]. ROAV was calculated by normalizing each OAV to the maximum OAV in the same sample. Therefore, ROAV ≥ 1 indicates that a compound contributes at least 1% of the maximum OAV in that sample; it does not indicate that the compound exceeds its absolute odor threshold. A total of 8 compounds with ROAV ≥ 1 are listed in Table 2.

β-Damascenone (ROAV = 100) served as the reference aroma compound in all samples, imparting a complex bouquet of apple, rose, and honey notes to the wines. Esters constituted the major contributors to aroma across all treatments, although their composition varied among groups. Ethyl octanoate exhibited the highest ROAV in Sc (10.25) and MS (11.93), followed by HS (1.43) and MHS (1.13), indicating that its contribution was primarily concentrated in fermentation systems dominated by *S. cerevisiae*. Ethyl hexanoate contributed consistently high values across all treatments (Sc 4.13, MS 4.09, HS 2.83, MHS 4.58), serving as a common component underlying the fruity base of jujube wines. Isoamyl acetate attained ROAV ≥ 1 only in MS (1.09) and MHS (1.41) groups, suggesting its relatively high within-sample aroma contribution in these two samples. Furthermore, benzeneacetaldehyde conferred honey-like notes to Hu (8.33), MS (13.27), and HS (5.12), while γ-decalactone (peach-like) had an ROAV > 1 only in the Mp group (1.10) and, together with phenethyl alcohol (1.61), shaped its peach-floral profile. The MHS samples exhibited a composite fruity–floral character, dominated by ethyl hexanoate (4.58) and isoamyl acetate (1.41), complemented by the rose-like floral note from phenethyl alcohol (1.51). In the CK samples, all compounds except β-damascenone exhibited ROAV < 1, indicating a relatively simple aroma-active profile.

#### 3.4.3. Partial Least Squares Discriminant Analysis (PLS-DA)

To identify the differential aroma compounds across the different treatment groups, a PLS-DA model was established (Figure S1a). The goodness-of-fit (R^2^Y) and predictive ability (Q^2^) of the model were 0.992 and 0.971, respectively, indicating good model performance. In the PLS-DA plot, the CK group was clearly separated from the fermented groups, and the single-culture fermentation groups were also clearly separated from the mixed-culture fermentation groups. This reflected the significant differences in the volatile compound composition of these groups. As shown in Figure S1b, after 200 permutation tests, the Q^2^ values of the random models remained consistently lower than the R^2^ values, and the intercept was below zero. This confirmed the validity of the model and the absence of overfitting. Thereafter, variable importance in projection (VIP) values were calculated. The VIP value reflects the contribution of a variable to the explanatory and predictive power of the model; the higher the VIP value, the greater the role of the variable in distinguishing among different sample categories [42]. In the present study, 26 compounds with VIP values > 1 were identified, including ethyl acetate, ethyl hexanoate, isoamyl alcohol, phenylethyl alcohol, ethyl decanoate, ethyl octanoate, and isoamyl acetate (Figure S1c). These emerged as the key volatile components for differentiating among the seven groups of samples.

### 3.5. Sensory Profile Analysis

**Figure 7:**
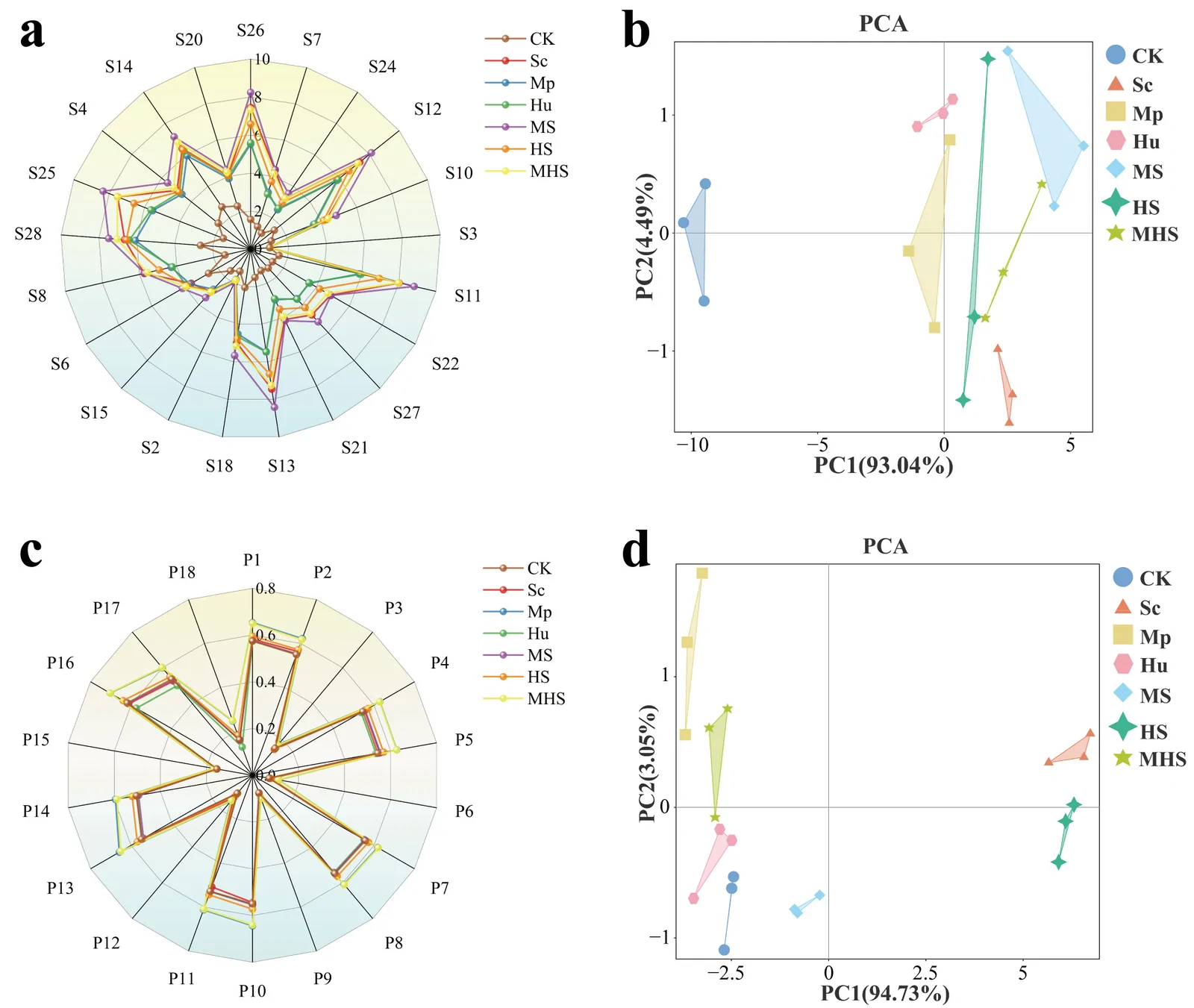
Electronic nose and electronic tongue analysis of different jujube wines. (**a**) Radar plot of electronic nose sensor responses; (**b**) PCA score plot of electronic nose data; (**c**) radar plot of electronic tongue response variables; (**d**) PCA score plot of electronic tongue data. CK, unfermented jujube juice; Sc, *S. cerevisiae* MST; Mp, *M. pulcherrima* HJ79; Hu, *H. uvarum* TD74; MS, sequential inoculation of *M. pulcherrima* HJ79 followed by *S. cerevisiae* MST; HS, sequential inoculation of *H. uvarum* TD74 followed by *S. cerevisiae* MST; MHS, simultaneous inoculation of *M. pulcherrima* HJ79 and *H. uvarum* TD74 followed by *S. cerevisiae* MST.

Combined electronic nose and electronic tongue analysis enabled the rapid and objective assessment of the aroma and taste attributes of jujube wines produced using different inoculation strategies. As illustrated in Figure 7a, the aroma profile of the CK group was relatively neutral, and the samples lacked distinct characteristic odors. In contrast, fermented samples produced markedly enhanced responses for sensors S11, S12, S13, S25, S26, S27, and S28, which are nominally responsive to volatile compounds including alkanes, esters, alcohols, and ketones. Such signal variations were in accordance with the aroma changes revealed by GC-MS analysis. The E-nose system effectively discriminated between CK and fermented samples. On PCA (Figure 7b), the mixed-culture fermentation groups clustered tightly along the PC1 axis, which indicated that they had a similar overall aromatic profile. However, these groups were clearly separated from the Sc group along the PC2 axis, likely due to the metabolic contributions of the non-*Saccharomyces* yeasts. The Mp and Hu groups clustered together in the PCA plot but were clearly separated from all other groups, indicating that these samples had a unique odor profile distinct from that of the other groups.

The electronic tongue data for different jujube wines are presented in Figure 7c,d (response variables P1 to P18). The radar plot showed that all treatment groups had largely similar response profiles across the P1–P18 response variables, with higher responses on response variables P1, P2, P4, P5, and P16 and lower responses on response variables such as P3, P6, and P9. Inter-group comparisons, however, revealed that the MHS and Mp groups exhibited slightly higher response values for some response variables, whereas the CK group displayed overall lower responses, suggesting a less distinct taste profile. In PCA analysis, the cumulative contribution of PC1 and PC2 to the overall variance was 97.78% (PC1: 94.73%, PC2: 3.05%). The Mp and MHS groups were located within the second quadrant; the CK, Hu, and MS groups clustered in the third quadrant; and the Sc and HS groups were distributed across the region toward the right. These results demonstrated that the inoculation strategy significantly altered the taste fingerprint of jujube wine.

### 3.6. Sensory Evaluation Results

**Figure 8:**
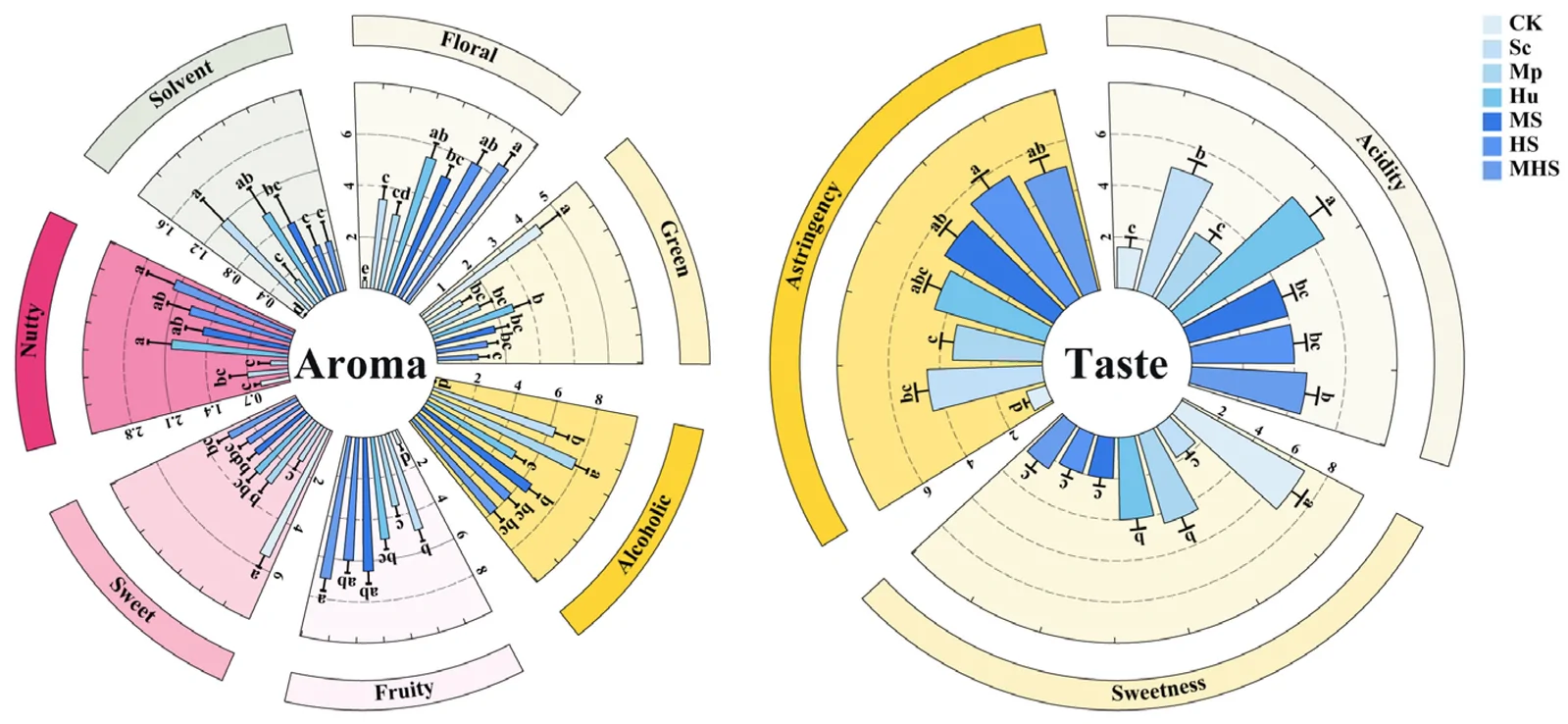
Sensory evaluation scores of jujube wine samples prepared using different inoculation strategies. Data are presented as mean ± SD from 10 trained assessors (*n* = 10). Different letters indicate significant differences among treatments (one-way ANOVA followed by Duncan’s multiple range test, *p* < 0.05). CK, unfermented jujube juice; Sc, *S. cerevisiae* MST; Mp, *M. pulcherrima* HJ79; Hu, *H. uvarum* TD74; MS, sequential inoculation of *M. pulcherrima* HJ79 followed by *S. cerevisiae* MST; HS, sequential inoculation of *H. uvarum* TD74 followed by *S. cerevisiae* MST; MHS, simultaneous inoculation of *M. pulcherrima* HJ79 and *H. uvarum* TD74 followed by *S. cerevisiae* MST.

The sensory evaluation results are presented in Figure 8. The Sc group received higher sensory scores for “alcoholic notes,” with low scores for “floral” and “fruity” aroma attributes, resulting in a relatively thin and simple overall aroma profile. Although the Hu and Mp groups scored relatively high on the “green note” and “sweetness” parameters, these sensory characteristics reflected the incomplete fermentation of the wine, which compromised overall quality. In contrast, the mixed-culture fermentation strategies appeared to improve aroma quality. The HS group achieved a relatively high score for the “floral” attribute, which was closely associated with the high contents of phenylethyl alcohol and terpenoids in this group. Meanwhile, the MS group stood out for its “fruity aroma.” With regard to mouthfeel, significant differences in the balance of “astringency,” “acidity,” and “sweetness” were observed among the treatment groups. Compared with the Sc group, the mixed-culture fermentation groups exhibited better “astringency” control and a more harmonious balance of sweetness and acidity. This prevented unpleasant sharpness on any single mouthfeel dimension. Of note, the MHS group showed the highest scores among the groups for the floral, fruity, and nutty attributes, relatively low scores for the green and solvent notes, and moderate acidity and sweetness, contributing to a more balanced sensory profile.

## 4. Conclusions

The sequential inoculation of non-*Saccharomyces* yeasts with *S. cerevisiae* effectively increased the total phenolic and total flavonoid contents of jujube wine, improved its antioxidant activity, and significantly enhanced its aromatic complexity. In comparison with single-culture fermentation using *S. cerevisiae*, multi-strain sequential inoculation enabled the production of higher levels of esters and alcohols, imparting pronounced floral and fruity characteristics to the wine. As a result, these wines received the most favorable sensory profile scores, particularly for floral and fruity notes. Our study confirmed that the inoculation of more than one non-*Saccharomyces* yeast is a viable strategy for enhancing the aroma diversity and quality of jujube wine. These findings provide a theoretical basis for evaluating the design of starter cultures and regulating flavor quality during jujube wine production. Nevertheless, this study has limitations. The specific enzymatic and regulatory mechanisms underlying the enhanced phenolic and volatile profiles in mixed-culture fermentations remain unclear. In addition, the 2-day delay between non-*Saccharomyces* and *S. cerevisiae* inoculation and the inoculation ratios were fixed and were not optimized in this study. Further multi-omics investigations, comparisons of different delay times and inoculation ratios, and pilot-scale validations are required to confirm the industrial applicability of this sequential inoculation strategy.

## Figures and Tables

**Table 1:** Effects of different inoculation methods on the physicochemical characteristics of jujube wine.

	CK	Sc	Mp	Hu	MS	HS	MHS
Reducing sugar (g/L)	186.43 ± 2.94 ^a^	6.10 ± 1.35 ^e^	117.32 ± 2.52 ^b^	110.98 ± 2.37 ^c^	11.69 ± 2.11 ^d^	12.20 ± 2.17 ^d^	7.20 ± 1.10 ^e^
Total acidity (g/L)	3.98 ± 0.03 ^d^	4.50 ± 0.10 ^c^	6.75 ± 0.13 ^a^	6.00 ± 0.07 ^b^	6.00 ± 0.23 ^b^	6.00 ± 0.18 ^b^	6.75 ± 0.09 ^a^
pH	4.00 ± 0.01 ^a^	3.73 ± 0.02 ^b^	3.62 ± 0.02 ^e^	3.61 ± 0.01 ^e^	3.71 ± 0.01 ^c^	3.67 ± 0.01 ^d^	3.71 ± 0.03 ^c^
Ethanol (% vol)		12.57 ± 0.38 ^a^	4.02 ± 0.21 ^f^	5.37 ± 0.31 ^e^	10.74 ± 0.22 ^d^	11.27 ± 0.30 ^c^	11.9 ± 0.18 ^b^
Total phenolic (mg GAE/L)	1441.24 ± 9.72 ^e^	1465.48 ± 12.01 ^e^	1463.25 ± 13.67 ^e^	1501.85 ± 8.90 ^d^	1536.83 ± 10.57 ^c^	1763.59 ± 17.82 ^b^	1917.12 ± 11.53 ^a^
Total flavonoid (mg RE/L)	94.37 ± 0.28 ^e^	99.35 ± 0.40 ^d^	102.75 ± 1.37 ^bc^	101.81 ± 0.64 ^c^	104.14 ± 1.92 ^b^	102.3 ± 1.15 ^bc^	115.35 ± 0.28 ^a^
L^*^	65.65 ± 0.20 ^b^	68.56 ± 0.31 ^a^	70.11 ± 1.74 ^a^	70.49 ± 1.85 ^a^	69.12 ± 1.89 ^a^	71.56 ± 1.90 ^a^	71.44 ± 1.66 ^a^
a^*^	3.51 ± 0.06 ^a^	1.15 ± 0.04 ^b^	0.65 ± 0.03 ^c^	−0.11 ± 0.08 ^f^	0.76 ± 0.28 ^c^	0.18 ± 0.06 ^e^	0.43 ± 0.01 ^d^
b^*^	34.72 ± 0.17 ^a^	28.20 ± 0.23 ^b^	27.91 ± 0.84 ^b^	25.68 ± 1.00 ^c^	26.16 ± 0.91 ^c^	23.71 ± 0.86 ^d^	27.01 ± 0.87 ^bc^
*△* E^*^		7.52 ± 0.30 ^b^	8.67 ± 1.58 ^b^	10.91 ± 1.71 ^ab^	9.69 ± 1.59 ^b^	12.98 ± 1.55 ^a^	10.15 ± 1.58 ^b^

Note: Different letters indicate significant differences among treatments (Duncan’s test, *p* < 0.05). Data are presented as mean ± SD (*n* = 3). CK, unfermented jujube juice; Sc, *S. cerevisiae* MST; Mp, *M. pulcherrima* HJ79; Hu, *H. uvarum* TD74; MS, sequential inoculation of *M. pulcherrima* HJ79 followed by *S. cerevisiae* MST; HS, sequential inoculation of *H. uvarum* TD74 followed by *S. cerevisiae* MST; MHS, simultaneous inoculation of *M. pulcherrima* HJ79 and *H. uvarum* TD74 followed by *S. cerevisiae* MST.

**Table 2:** ROAV analysis of volatile compounds in different jujube wines based on semi-quantitative GC-MS data.

Compounds	Aroma Description	Threshold (μg/L) ^α^	Semi Quantitative OAV							ROAV/%						
			CK	Sc	Mp	Hu	MS	HS	MHS	CK	Sc	Mp	Hu	MS	HS	MHS
Isoamyl acetate	Fruity	30	-	4.36 ± 0.17 ^c^	5.47 ± 0.18 ^bc^	5.20 ± 0.28 ^b^	6.43 ± 0.22 ^b^	5.10 ± 0.21 ^c^	11.04 ± 1.40 ^a^	-	0.81 ± 0.04 ^c^	0.57 ± 0.02 ^d^	0.91 ± 0.15 ^c^	1.09 ± 0.11 ^b^	0.86 ± 0.03 ^c^	1.41 ± 0.18 ^a^
Ethyl hexanoate	Apple	14	0.02 ± 0.00 ^d^	22.30 ± 1.54 ^b^	2.24 ± 0.10 ^d^	1.53 ± 0.24 ^d^	24.12 ± 1.10 ^b^	16.87 ± 0.65 ^c^	35.57 ± 3.43 ^a^	0.01 ± 0.00 ^d^	4.13 ± 0.37 ^a^	0.23 ± 0.01 ^c^	0.26 ± 0.02 ^c^	4.09 ± 0.47 ^a^	2.83 ± 0.10 ^b^	4.58 ± 0.87 ^a^
Ethyl octanoate	Caramel, fruity	5	-	55.32 ± 4.37 ^b^	-	0.76 ± 0.11 ^d^	70.47 ± 3.64 ^a^	8.55 ± 0.54 ^c^	8.94 ± 1.42 ^c^	-	10.25 ± 0.97 ^b^	-	0.13 ± 0.01 ^d^	11.93 ± 1.40 ^a^	1.43 ± 0.01 ^c^	1.13 ± 0.05 c^d^
Ethyl decanoate	Fruity	20	-	6.03 ± 0.30 ^b^	-	0.39 ± 0.18 ^e^	6.86 ± 0.46 ^a^	2.11 ± 0.15 ^c^	1.18 ± 0.21 ^d^	-	1.12 ± 0.07 ^a^	-	0.06 ± 0.01 ^c^	1.16 ± 0.16 ^a^	0.35 ± 0.00 ^b^	0.15 ± 0.01 ^c^
γ-Decalactone	Peach	0.7	-	-	10.53 ± 0.28 ^a^	-	2.64 ± 0.33 ^c^	2.57 ± 0.31 ^c^	3.98 ± 0.41 ^b^	-	-	1.10 ± 0.04 ^a^	-	0.45 ± 0.05 ^b^	0.43 ± 0.03 ^b^	0.51 ± 0.07 ^b^
Phenethyl alcohol	Rose	45	-	4.35 ± 0.36 ^cd^	15.36 ± 3.28 ^a^	4.05 ± 0.54 ^cd^	2.99 ± 0.23 ^d^	6.56 ± 0.59 ^c^	11.79 ± 1.27 ^b^	-	0.81 ± 0.08 ^c^	1.61 ± 0.35 ^a^	0.70 ± 0.09 ^cd^	0.51 ± 0.05 ^d^	1.10 ± 0.03 ^b^	1.51 ± 0.16 ^a^
Benzeneacetaldehyde	Honey	4	-	-	-	47.45 ± 1.85 ^b^	78.66 ± 4.49 ^a^	30.57 ± 1.19 ^c^	-	-	-	-	8.33 ± 1.51 ^b^	13.27 ± 0.55 ^a^	5.12 ± 0.21 ^c^	-
β-Damascenone	Apple, rose aroma, honey	0.05	274.11 ± 23.13 ^d^	540.53 ± 14.92 ^c^	955.21 ± 8.33 ^a^	587.07 ± 141.79 ^c^	593.49 ± 39.64 ^c^	597.93 ± 43.49 ^c^	787.52 ± 94.39 ^b^	100	100	100	100	100	100	100

Note: “-“ indicates not detected. ^α^ Odor thresholds were compiled from the literature (values for water or hydro-alcoholic solutions commonly used in wine OAV studies) [21,40,41]. ROAV analysis of volatile compounds in different jujube wines based on semi-quantitative data. Data are presented as mean ± SD (*n* = 3). Different letters in the same row indicate significant differences among treatments (Duncan’s multiple range test, *p* < 0.05). ROAV_i_ = (OAV*_i_*/OAV_max_) × 100, where OAV*_i_* = C*_i_*/T*_i_*, C*_i_* is the semi-quantitative concentration, and T*_i_* is the odor threshold from the literature. ROAV ≥ 1 indicates a relatively important aroma contributor within each sample and should not be interpreted as exceeding the absolute odor threshold. CK, unfermented jujube juice; Sc, *S*. *cerevisiae* MST; Mp, *M*. *pulcherrima* HJ79; Hu, *H*. *uvarum* TD74; MS, sequential inoculation of *M. pulcherrima* HJ79 followed by *S. cerevisiae* MST; HS, sequential inoculation of *H. uvarum* TD74 followed by *S. cerevisiae* MST; MHS, simultaneous inoculation of *M. pulcherrima* HJ79 and *H. uvarum* TD74 followed by *S. cerevisiae* MST.

## Data Availability

The original contributions presented in the study are included in the article/Supplementary Materials, further inquiries can be directed to the corresponding authors.

## Supplementary Materials

The following supporting information can be downloaded at https://www.mdpi.com/article/10.3390/foods15203604/s1. Figure S1: PLS-DA of volatile compounds in different jujube wine samples. (a) Score plot; (b) Permutation test plot; (c) VIP plot. Table S1: Sensors in the electronic nose system and their target volatile compounds. Table S2: Definitions and reference standards of the sensory attributes for jujube wine. Table S3: Two-way repeated-measures ANOVA results for time-course fermentation data. Table S4: Semi-quantitative contents of volatile compounds in jujube wines under different fermentation strategies.
