# Supplementary material for: Influence of Fermentation Methods on Jujube Wine Quality: A Volatile Metabolomics-Based Investigation
Source: Foods. 2026 Oct 10;15(20):3604. doi: 10.3390/foods15203604 (PMC13703251; doi:10.3390/foods15203604)
Supplement: Supplementary file 1 [file foods-15-03604-s001.zip › foods-4571651-supplementary.pdf]

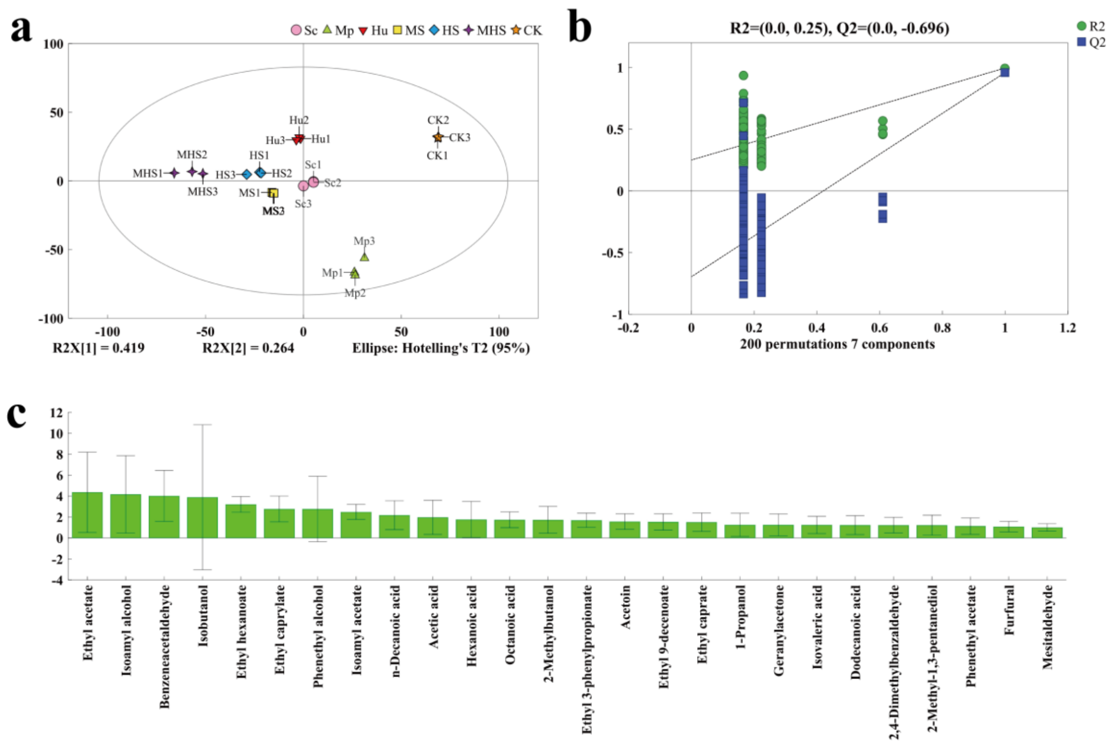

Figure S1. PLS-DA of volatile compounds in different jujube wine samples. (a) Score plot; (b) Permutation test plot; (c) VIP plot

Table S1 Sensors in the electronic nose system and their target volatile compounds

Sensors	Substances for sensing
S1	Propane, smoke, etc
S2	Carbon-containing substances: alcohol, smoke, isobutane, formaldehyde, etc.
S3	Hydrogen
S4	Sulfides
S5	Amine-containing substances: ammonia, amines, etc.
S6	Aldehydes and ketones: toluene, acetone, ethanol, hydrogen, etc.
S7	Short-chain alkanes, combustible gases, etc.
S8	Liquefied gas
S9	Alkanes, alcohols, ketones, etc.: toluene, formaldehyde, benzene, alcohol, acetone, etc.
S10	Hydrogen-containing gases
S11	Alkanes, carbon monoxide, etc.
S12	Some organic solvents: liquefied gas, methane series
S13	Short-chain alkanes
S14	Short-chain alkanes: methane, fuel gas, smoke, etc.
S15	Amine-containing: ammonia, amines, etc.
S16	Sulfides: hydrogen sulfide
S17	Hydrogen-containing substances
S18	Aromatic hydrocarbons, aliphatic hydrocarbons, alicyclic hydrocarbons, halogenated hydrocarbons, ethers, esters, glycol derivatives, acetonitrile, pyridine, phenol, etc.
S19	Alcohols, ketones, aldehydes, aromatic compounds, etc.
S20	Alkanes, alkenes, aromatic hydrocarbons, etc.: methane, biogas, natural gas, etc.
S21	Short-chain alkanes: combustible gases, etc.
S22	Phenol, ketones, ethyl acetate, cyclohexanone, chlorobenzene, methylbenzene, ethers, etc.
S23	Alkanes, alkenes, aromatic hydrocarbons, etc.
S24	Alkanes, alkenes, liquefied gas, combustible gases, etc.
S25	Alkanes, alcohol, natural gas, smoke, etc.
S26	Some esters, ketones, aromatic hydrocarbons, aliphatic hydrocarbons, alicyclic hydrocarbons, halogenated hydrocarbons
S27	Sulfides, nitrides, carbides, hydrocarbons, and nitrogen-oxygen compounds, etc.: flue gas, cooking odors, etc.
S28	Short-chain alkanes: methane, fuel gas, etc.

Table S2 Definitions and reference standards of the sensory attributes for jujube wine

Attribute	Definition	Reference Standard
Aroma		
Floral	Similar to floral notes of rose, lily, osmanthus, etc.	10 μg/L phenylethanol in aqueous solution (5.0)
Fruity	Similar to fruity notes of ripe apple, peach, apricot	Mixed solution of 20 μg/L isoamyl acetate and 10 μg/L ethyl hexanoate (5.0)）
Sweet	Similar to caramel and honey	5 g/L honey aqueous solution (4.0)
Nutty	Similar to nutty notes of almond and cherry pit	1 mg/L benzaldehyde aqueous solution (5.0)
Alcoholic	Odor of ethanol volatilization	10% (v/v) edible alcohol aqueous solution (5.0)
Green	Similar to vegetative/green notes of grass, leaves, green pepper	2% (w/v) cis-3-hexenol solution (5.0)
Solvent	Chemical pungent odor similar to nail polish, glue, organic solvents	50 mg/L ethyl acetate aqueous solution (5.0)
Taste		
Acidity	Sour taste on the sides of the tongue caused by tartaric acid, citric acid, etc.	1 g/L citric acid aqueous solution (3.0); 2 g/L (6.0)
Sweetness	Sweet taste on the tip of the tongue from sucrose, etc.	5 g/L sucrose aqueous solution (3.0); 20 g/L (7.0)
Astringency	Astringent and rough sensation on oral mucosa caused by tannins, etc.	0.5 g/L tannic acid aqueous solution (4.0); 1.0 g/L (7.0)

Table S3 Two-way repeated-measures ANOVA results for time-course fermentation data

Figure	Panel	Variable	Effect	df	F value	*p* value
Figure 1	1a	Yeast population (mono-culture)	Treatment	2, 78	413.1	<0.001
			Time	12, 78	562.4	<0.001
			Treatment × Time	24, 78	8.1	<0.001
	1b	Yeast population (MS)	Treatment	1, 40	994.5	<0.001
			Time	9, 40	167.7	<0.001
			Treatment × Time	9, 40	208.8	<0.001
	1c	Yeast population (HS)	Treatment	1, 40	269.0	<0.001
			Time	9, 40	134.4	<0.001
			Treatment × Time	9, 40	177.5	<0.001
	1d	Yeast population (MHS)	Treatment	2, 66	275.8	<0.001
			Time	10, 66	308.1	<0.001
			Treatment × Time	20, 66	113.8	<0.001
Figure 2		β-Glucosidase activity	Treatment	5, 84	2216.5	<0.001
			Time	6, 84	2770.2	<0.001
			Treatment × Time	30, 84	115.0	<0.001
Figure 3	3a	Soluble solids content	Treatment	5, 156	4970.1	<0.001
			Time	12, 156	9056.5	<0.001
			Treatment × Time	60, 156	113.1	<0.001
	3b	Reducing sugar	Treatment	5, 84	4059.7	<0.001
			Time	6, 84	11301.5	<0.001
			Treatment × Time	30, 84	311.2	<0.001
	3c	Ethanol content	Treatment	5, 84	1322.1	<0.001
			Time	6, 84	3514.2	<0.001
			Treatment × Time	30, 84	94.7	<0.001

Notes: df, degrees of freedom (numerator, denominator). All time-course data were analyzed by two-way repeated-measures ANOVA with inoculation treatment and fermentation time as fixed factors. Balanced time points were used for each analysis: Figure 1a, days 0–12; Figure 1b and 1c, days 2–11; Figure 1d, days 2–12; Figure 2 and Figure 3b/c, days 0, 2, 4, 6, 8, 10, 12; Figure 3a, days 0–12. n = 3 independent fermentation replicates for all analyses. All effects were statistically significant ($p < 0.001$).

Table S4 Semi-quantitative contents of volatile compounds in jujube wines under different fermentation strategies

Compounds	CAS	Retention index (RI)	Sc	Mp	Hu	MS	HS	MHS	CK
**Esters (51)**									
Ethyl acetate	141-78-6	897.3	201.92±14.04d	ND	1474.89±78.86b	320.63±20.05d	1219.4±85.9c	2035.95±205.74a	0.89±0.11e
Ethyl butyrate	105-54-4	1040.3	8.6±0.85b	ND	0.72±0.22d	10.65±0.61a	7.19±0.47c	8.74±1.38b	ND
Ethyl valerate	539-82-2	1141.1	10.09±0.42b	ND	ND	14.9±3.14a	11.97±1.75b	15.61±1.4a	ND
Isoamyl acetate	123-92-2	1125.7	130.71±5.21d	164.13±5.44bc	155.89±8.49cd	192.92±6.53b	153.14±6.44cd	331.33±42.05a	ND
Ethyl hexanoate	123-66-0	1241	312.18±21.56b	31.37±1.44d	21.36±3.4d	337.7±15.37b	236.21±9.04c	498.02±47.96a	0.27±0.04
Ethyl heptanoate	106-30-9	1341.3	28.88±1.89b	ND	3.29±0.37d	29.9±1.54ab	23.04±1.21c	33.84±6.18a	ND
Ethyl octanoate	106-32-1	1445.6	276.61±21.86b	ND	3.82±0.53d	352.37±18.18a	42.73±2.71c	44.72±7.12c	ND
Ethyl nonanoate	123-29-5	1542.3	2.37±0.52a	ND	ND	2.52±0.19a	1.19±0.36b	0.89±0.03b	ND
Methyl decanoate	110-42-9	1605.8	1.73±0.64a	ND	ND	1.08±0.21b	ND	ND	ND
Ethyl decanoate	110-38-3	1644.8	120.69±5.94b	ND	7.72±3.52e	137.17±9.13a	42.24±3.08c	23.55±4.21d	ND
Ethyl benzoate	93-89-0	1689.3	2.22±0.32a	2.3±0.24a	0.81±0.52d	2.32±0.2a	1.17±0.09bc	1.61±0.13b	ND
Ethyl phenylacetate	101-97-3	1806.2	1.64±0.06b	0.77±0.23c	0.92±0.44c	2.17±0.1a	1.54±0.18b	1.41±0.1b	ND
Phenethyl acetate	103-45-7	1836.9	28.38±2.86d	0.87±1.5e	101.68±12.76b	29.61±1.27d	87.5±4.75c	145.68±9.41a	ND
Isobutyl acetate	110-19-0	1018.4	ND	38.96±2.94a	ND	ND	ND	11.86±0.74b	ND
Isoamyl propionate	105-68-0	1195.1	2.24±0.47a	0.75±0.05b	ND	2.01±0.06a	ND	ND	ND
Hexyl acetate	142-92-7	1264	ND	0.49±0.06b	ND	0.45±0.09b	ND	1.19±0.12a	ND
Ethyl 7-octenoate	35194-38-8	1495.1	12.66±0.79a	ND	5.49±7.27ab	12.51±3.23a	10.27±2.22a	6.57±5.93ab	ND
Ethenylformate	692-45-5	842	14.55±0.64a	10.58±1.12b	5.64±0.68d	9.11±1.35bc	8.53±1.26c	7.24±1.74cd	ND
Ethyl 4-hexenoate	34495-74-4	1238	3.07±0.03b	ND	ND	3.51±0.86b	ND	5.53±0.77a	ND
1,2-Dimethyl-2-cyclopentene-1-carboxylic acid	64701-60-6	1924.3	ND	21.6±2.99a	1.8±0.08b	ND	ND	ND	ND
Ethyl hex-2-enoate	1552-67-6	1355	3.6±0.45a	ND	ND	3.28±0.52a	3.24±0.96a	2.22±0.84b	ND
(E)-Ethyl 3-hexenoate	26553-46-8	1309.2	3.54±0.59a	ND	ND	4.45±1.55a	4.62±1.48a	4.7±2.34a	ND

Ethyl (E)-2-heptenoate	54340-72-6	1460.4	1.42±0.64c	ND	ND	1.64±0.16c	2.86±0.67b	5.22±0.64a	ND
Ethyl 3-hydroxybutyrate	5405-41-4	1446	0.72±0.28a	ND	ND	0.7±0.09a	0.45±0.1b	0.48±0.07b	ND
Furfuryl acetate	623-17-6	1472	0.68±0.16d	ND	10.05±0.11b	ND	4.44±0.26c	11.57±0.75a	ND
Diethyl succinate	123-25-1	1691	5.86±0.42b	ND	ND	9.93±0.83a	ND	ND	ND
Ethyl 9-decenoate	67233-91-4	1700.5	88.18±5.6b	ND	ND	103.1±4.64a	11.02±0.91c	7.49±0.51c	ND
Ethyl dodecanoate	106-33-2	1849.2	5.43±0.71a	ND	2.03±0.15c	4.06±0.57b	4.07±0.49b	3.55±0.91b	ND
Ethyl 3-phenylpropionate	2021-28-5	1904.7	160.41±6.57a	11.42±0.06c	18.67±2.94c	154.54±9.31a	112.56±5.58b	108.2±7.77b	0.32±0.02d
Ethyl 3-hydroxyoctanoate	7367-90-0	1718	0.76±0.16b	ND	ND	1.05±0.15a	ND	ND	ND
γ-Octalactone	104-50-7	1949.7	0.91±0.11bcd	3.31±0.71a	0.46±0.03cd	1.03±0.36bc	0.98±0.25bc	1.37±0.2b	0.32±0.02d
Diethyl azelate	624-17-9	2013	1.15±0.04a	ND	ND	0.76±0.1b	ND	ND	ND
Methyl hexadecanoate	112-39-0	2229.5	0.89±0.09a	ND	0.34±0.03c	0.75±0.13b	ND	ND	ND
2-(dodecyloxy)ethanol	4536-30-5	2186.2	1.66±0.15a	0.88±0.12c	0.68±0.02d	1.59±0.1ab	1.48±0.15ab	1.4±0.08b	ND
Ethyl cetylate	628-97-7	2258.5	2.85±0.04a	1.03±0.3b	0.93±0.06b	3±0.06a	2.56±0.32a	2.62±0.44a	ND
Propyl hexanoate	626-77-7	1343.1	0.47±0.02b	ND	ND	0.63±0.07a	0.4±0.02b	0.61±0.16a	ND
Methyl acetate	79-20-9	881.3	ND	0.76±0.13b	8.61±0.89a	ND	ND	ND	ND
δ-Dodecalactone	713-95-1	2092.6	ND	5.48±0.75a	ND	ND	ND	ND	ND
Ethyl lactate	97-64-3	1350.4	ND	0.45±0.17c	0.55±0.01c	1.11±0.15b	1.36±0.19a	ND	ND
γ-Caprolactone	695-06-7	1614.7	ND	1.57±0.42a	0.79±0.13b	ND	ND	ND	0.81±0.04b
Ethyl (E)-hept-3-enoate	54340-71-5	1387.2	1.49±0.44a	ND	ND	ND	ND	ND	ND
Butyl acetate	123-86-4	1078	ND	ND	1.87±0.73b	ND	ND	3.8±0.74a	ND
Propyl octanoate	624-13-5	1536.1	ND	ND	ND	0.53±0.11a	ND	ND	ND
γ-Decalactone	706-14-9	2791.4	ND	7.37±0.19a	ND	1.85±0.23c	1.8±0.22c	2.79±0.28b	ND
3-Methylbutyl octanoate	2035-99-6	1691.2	ND	ND	ND	1.28±0.09a	ND	ND	ND
Pentyl acetate	628-63-7	1172.8	ND	ND	ND	ND	1.53±0.2a	ND	ND
2,3-Hexadienoic acid methyl ester	110318-09-7	1488	ND	ND	ND	ND	3.08±0.01a	3.31±0.65a	ND
Pentyl propanoate	624-54-4	1266.9	ND	ND	ND	ND	ND	4.75±0.15a	ND

Heptyl acetate	112-06-1	1407.5	ND	ND	ND	ND	1.54±0.5a	ND	ND
Isopropenyl acetate	108-22-5	967	ND	ND	ND	ND	ND	ND	1.42±0.13a
Methyl 2-furoate	611-13-2	1425	ND	ND	ND	ND	ND	ND	1.39±0.12a
**Alcohols (44)**									
Phenylethyl alcohol	60-12-8	1930.4	195.87±16cd	691.16±147.72a	182.15±24.36cd	134.57±10.29d	295.08±26.48c	530.57±56.96b	0.08±0.03e
1-Propanol	71-23-8	1040.3	16.41±1.95b	10.69±1.98c	ND	33.94±3.95a	32.1±2.82a	18.39±1.53b	ND
1-Butanol	71-36-3	1148.8	ND	3.29±0.27a	ND	ND	ND	ND	ND
Isobutyl alcohol	78-83-1	1095.1	28.32±4.43c	1386.9±40.36a	34.8±7.65c	53.12±2.97bc	52.79±2.09bc	66.4±3.55b	ND
2-Methyl-1-butanol	137-32-6	1209	173.21±18.47bc	289.38±70.7a	119.65±6.74c	284.4±1.66a	220.21±17.33ab	247.16±67.91a	ND
(R,R)-2,3-Butanediol	24347-58-8	1589.7	3.32±0.57ab	3.95±0.99a	1.83±0.9c	1.53±0.23c	2.35±0.62bc	2.17±0.91bc	ND
1-Pentanol	71-41-0	1236	ND	1.84±0.03a	1.09±0.02b	1.49±0.5ab	ND	1.67±0.53a	ND
3-Methyl-1-butanol	123-51-3	1207.3	1473.25±77.48c	1448.1±45.42c	997.58±28.28d	1919.14±12.94a	1788.86±49.37b	1931.93±111.45a	0.69±0.02e
3-Methyl-1-pentanol	589-35-5	1332.1	1.28±0.45b	0.93±0.07bc	0.39±0.07c	1.48±0.18ab	1.15±0.1bc	2.16±0.96a	ND
1-Hexanol	111-27-3	1355	5.54±1.07c	6.92±0.2ab	4.07±0.77d	7.86±0.32a	6.56±0.2bc	ND	ND
1-Octanol	111-87-5	1563.3	4.42±0.18b	ND	2.34±0.21c	1.63±0.57c	4.02±1.15b	7.47±1.06a	ND
2-Ethylhexanol	104-76-7	1490.2	12.49±1.21a	2.12±0.36d	4.1±0.16c	3.72±0.85c	6.22±0.14b	7.38±0.87b	1.26±0.1d
1-Heptanol	111-70-6	1040.3	15.26±1.02a	1.63±0.04f	10.12±0.35d	7.71±0.16e	12.1±0.36c	14.3±0.45b	ND
3-Methyl-2-heptanol	31367-46-1	1347	ND	2.59±0.37a	ND	ND	ND	ND	ND
Benzyl alcohol	100-51-6	1898.3	ND	2.47±0.69a	0.89±0.13c	0.85±0.15c	ND	1.76±0.08b	0.4±0.02c
1-Decanol	112-30-1	1764.9	4.17±0.18a	ND	0.59±0.23c	3.8±0.01b	0.69±0.06c	0.61±0.08c	ND
Citronellol	106-22-9	1770.7	3.37±0.19b	ND	0.55±0.03d	1.46±0.19c	3.89±0.24a	3.52±0.44ab	ND
1-Dodecanol	112-53-8	1668.2	7.5±0.32a	3.12±0.44d	4.17±0.23c	6.26±0.44b	6.44±0.44b	6.3±0.18b	0.57±0.23e
1-Tetradecanol	112-72-1	1864.9	1.64±0.31bc	1.53±0.25c	2.1±0.32ab	1.8±0.22abc	2.2±0.16a	2.05±0.19ab	ND
Farnesol	4602-84-0	2359	3.26±0.28b	1.41±0.05d	1.53±0.05d	3.62±0.22b	4.3±0.52a	2.71±0.25c	ND
1-Octen-3-ol	3391-86-4	1430	ND	3.99±0.38b	7.41±5.32b	8.48±3.37ab	7.93±2.18ab	13.71±2a	ND
2-Octanol	123-96-6	1420.8	ND	1.25±0.35a	0.77±0.02b	ND	ND	ND	ND

4-Penten-1-ol	821-09-0	1170	0.98±0.02ab	1.34±0.05a	0.38±0.03c	0.61±0.02bc	0.74±0.29bc	0.96±0.48ab	ND
2-Tridecanol	1653-31-2	1841.9	2.3±0.2a	ND	ND	ND	ND	ND	ND
n-Tridecanol	112-70-9	1956	2.37±0.2a	ND	ND	ND	ND	ND	ND
1,9-Nonanediol	3937-56-2	1417.8	0.88±0.11b	ND	ND	1.08±0.13a	ND	ND	ND
n-Pentadecanol	629-76-5	1889.2	1.08±0.22ab	ND	0.77±0.07b	ND	0.97±0.19ab	1.19±0.19a	ND
2-Furanmethanol	98-00-0	1672.6	ND	5.15±0.93a	2.71±0.3b	2.93±0.2b	3.07±0.45b	3.13±0.75b	0.38±0.04c
L-α-Terpineol	10482-56-1	1704.3	ND	1.06±0.1a	ND	ND	ND	ND	ND
Nerolidol	40716-66-3	2050	2.67±0.28b	1.44±0.15c	0.45±0.12d	3.98±0.22a	1.65±0.17c	1.33±0.32c	ND
2-Methyl-1,3-pentanediol	149-31-5	1753.6	ND	ND	ND	23.26±3.97a	ND	ND	ND
4-Methyl-1-pentanol	626-89-1	1318	ND	ND	ND	0.61±0.12a	ND	ND	ND
2-Heptanol	543-49-7	1303.2	ND	ND	ND	0.53±0.01a	ND	ND	ND
5-Aminododecahydro-as-indacen-4-ol	57366-15-1	1389.2	15.93±2.06a	13.05±1a	6.97±0.98b	13.7±1.02a	15.28±3.21a	12.84±1.76a	0.6±0.02c
(5-Methyl-2-furyl) methanethiol	59303-05-8	1496	6.91±0.28b	ND	2.82±0.2d	4.35±0.66c	3.26±0.4d	8.69±0.1a	ND
2,3-Dihydro-6-trans-farnesol	20576-54-9	2145.8	2.32±0.07a	ND	ND	1.88±0.06b	0.9±0.05c	0.49±0.12d	ND
6-Methyl-5-hepten-2-ol	1569-60-4	1441.5	ND	1.84±0.08a	ND	0.98±0.34b	ND	2.11±0.05a	ND
cis-4-Hexen-1-ol	928-91-6	1372	ND	ND	0.61±0.07b	ND	0.97±0.15a	ND	ND
Geraniol	106-24-1	1842.9	ND	ND	12.61±1a	ND	ND	ND	ND
5-Methylnonan-5-ol	33933-78-7	1483	ND	ND	ND	0.99±0.01b	1.14±0.02a	ND	ND
2-Methyl-1-pentanol	105-30-6	1334.2	ND	ND	ND	ND	ND	0.32±0.02a	ND
6-Methyl-4-heptanol	21570-35-4	1361	ND	ND	ND	ND	4.54±0.25a	ND	ND
1,3-Ethoxypropanol	111-35-3	1540	ND	ND	4.13±0.1b	ND	6.53±0.2a	ND	ND
cis-Linalool oxide	5989-33-3	1466.8	ND	0.79±0.06a	0.67±0.11ab	ND	ND	0.58±0.06bc	0.49±0.02c
**Aldehydes (19)**									
Benzaldehyde	100-52-7	1547.5	0.61±0.24e	5.9±0.39b	3.51±0.27c	6.25±0.47b	2.67±0.11cd	1.83±0.17de	21.95±2.02a
Benzeneacetaldehyde	122-78-1	1667.1	ND	ND	189.81±7.4b	314.65±17.96a	122.27±4.74c	ND	ND

2-Methylbutanal	96-17-3	864.9	ND	ND	ND	ND	ND	ND	0.72±0.35a
Hexanal	66-25-1	1087.8	ND	ND	ND	ND	ND	ND	1.47±0.3a
Heptanal	111-71-7	1191.2	ND	ND	2.58±0.84b	5.47±0.1a	1.24±0.66c	ND	0.53±0.07c
Octanal	124-13-0	1277	ND	ND	3.43±0.19b	4.37±0.87a	1.62±0.58c	0.66±0.18d	0.29±0.15d
2,4-Dimethylbenzaldehyde	15764-16-6	1708	58.3±8.94ab	63.55±5.59a	48.2±9.93bc	52.22±4.69bc	42.52±3.46c	43.95±0.21c	18.98±0.89d
Decanal	112-31-2	1510.6	2.68±0.6a	1.05±0.17b	1.24±0.39b	3.39±0.21a	3.25±0.52a	2.98±0.51a	ND
γ-Amylbutyrolactone	104-61-0	2061.8	6.17±0.35bc	9.93±1.13a	4.89±0.61d	6.62±0.42bc	5.53±0.69cd	7±0.25b	0.35±0.02e
γ-Undecalactone	104-67-6	1937.1	1.59±0.08c	5.95±0.55a	2.82±0.61b	ND	ND	ND	ND
Nonanal	124-19-6	1405.9	ND	ND	7.03±0.17a	ND	ND	6.34±0.2b	ND
2-Methylbenzaldehyde	529-20-4	1681	1.04±0.01b	ND	ND	ND	ND	1.33±0.15a	ND
Mesitaldehyde	487-68-3	1756.2	ND	ND	ND	9.66±1.55b	11.92±1.73ab	12.6±0.96a	3.42±0.32c
10-Undecenal	112-45-8	1941	ND	ND	ND	0.87±0.41a	ND	ND	ND
(2E)-Heptenal	18829-55-5	1325.6	ND	ND	ND	ND	ND	ND	0.88±0.01a
(E)-2-Hexenal	6728-26-3	1222.4	ND	ND	ND	ND	ND	ND	0.4±0.27a
(E)-2-Octenal	2548-87-0	1427.8	ND	ND	ND	ND	ND	ND	0.52±0.02a
Furfural	98-01-1	1480.9	ND	ND	ND	ND	ND	ND	40.41±6.4a
5-Methyl furfural	620-02-0	1571	ND	ND	ND	ND	ND	ND	11.55±1.01a
**Ketones (10)**									
Damascenone	23696-85-7	1362	2.09±0.04b	3.64±0.16a	2.58±0.55b	1.99±0.4b	2.21±0.47b	2.25±0.37b	0.79±0.12c
β-Damascenone	23726-93-4	1843	27.03±0.75c	47.76±0.42a	29.35±7.09c	29.67±1.98c	29.9±2.17c	39.38±4.72b	13.71±1.16d
Acetophenone	98-86-2	1650.2	ND	ND	ND	ND	ND	ND	0.58±0.06a
Butyrophenone	495-40-9	1774.1	ND	ND	0.49±0.06a	ND	ND	ND	ND
6-Methyl-5-hepten-2-one	110-93-0	1345.8	2.39±0.32a	ND	0.86±0.45bc	ND	1.2±0.46b	0.73±0.55bc	0.38±0.03c
Geranylacetone	689-67-8	1867.6	17.21±0.5a	ND	ND	ND	ND	ND	ND
1-(1,3-Dimethyl-3-cyclohexen-1-yl)	51733-68-7	1620.9	ND	ND	2.5±0.12b	ND	ND	3.61±0.44a	ND

ethanone									
6-Methyl-3,5-heptadien-2-one	1604-28-0	1511	ND	ND	ND	ND	ND	ND	0.48±0.03a
2-Hydroxy-3-pentanone	5704-20-1	1338.6	ND	ND	3.38±0.25a	ND	ND	ND	ND
Acetoin	513-86-0	1294	ND	50.58±9.43a	48.88±13.88a	ND	ND	ND	3.02±0.91b
**Acids (26)**									
Benzoic acid	65-85-0	2443	ND	2.72±0.31a	1.95±0.45b	ND	ND	ND	0.71±0.08c
Dodecanoic acid	143-07-7	2490.9	2.48±0.55c	2.45±1.07c	34.34±1.42a	3.9±0.57bc	4.44±0.42b	3.86±0.61	2.91±1.3bc
Pentanoic acid	109-52-4	1731	1.18±0.11de	3.31±0.24b	2.35±0.17c	1.07±0.15e	1.49±0.19d	2.48±0.35c	4.79±0.16a
Nonanoic acid	112-05-0	2179.4	8.64±3.71bc	4.05±1.63c	19.57±3.79a	13.8±4.42ab	14.1±3.12ab	16.48±3.91a	5.22±0.23c
Octanoic acid	124-07-2	2068.5	111.61±14.5b	6.54±1.14d	54.07±11.03c	138.53±14.96a	44.02±5.81c	53.35±6.28c	11.38±0.35d
Hexanoic acid	142-62-1	1855.3	39.88±8.89bc	48.34±1.78b	60.44±6.61a	35.21±1.67c	23.73±3.42d	46.59±2.89b	47.03±1.12b
Decanoic acid	334-48-5	2280.3	45.35±9.15b	5.63±1.43c	133.88±24.34a	39.62±2.08b	51.33±2.95b	48.08±8.68b	15.5±1.48c
Acetic acid	64-19-7	1465.4	70.39±10.66cd	27.9±3.43e	157.63±38.21a	40.73±4.71de	96.5±10.2bc	118.88±17.68b	23.5±4.83e
Propanoic acid	79-09-4	1530	1.41±0.25a	1.66±0.13a	1.5±0.66a	1.49±0.26a	1.48±0.16a	1.62±0.3a	1.49±0.48a
Heptanoic acid	111-14-8	1962.6	7.74±2.1cd	ND	21.09±0.44a	3.61±0.29e	6.72±2.38d	11.35±2.24b	10.4±1.09bc
9-Decenoic acid	14436-32-9	2343.7	9.04±0.89a	ND	2.51±0.6c	7.74±0.91b	2.39±0.31c	2.63±0.2c	0.3±0.03d
Isovaleric acid	503-74-2	1683.8	ND	15.62±0.7c	8.85±1.66d	20.52±3.75b	19.71±4.37bc	26.09±1.86a	6.85±0.86d
Isobutyric acid	79-31-2	1579.1	1.48±0.2c	3.68±0.29a	1.88±0.29c	1.54±0.36c	1.64±0.21c	2.42±0.23b	0.61±0.2d
Isooctanoic acid	149-57-5	1826	ND	4.73±1.28ab	ND	3.59±1.32b	4.14±1.05ab	6.06±1.12a	ND
Butanoic acid	107-92-6	1620.2	ND	4.19±0.02b	ND	ND	ND	0.91±0.27c	4.66±0.29a
E-3-Decenoic acid	53678-20-9	2110	ND	4.22±0.58a	ND	ND	ND	ND	0.19±0.01b
Tetradecanoic acid	544-63-8	2463.7	ND	1.04±0.27a	1.18±0.24a	ND	ND	ND	ND
3-Methylvaleric acid	105-43-1	1714	6±0.16a	ND	ND	ND	ND	ND	ND
E-2-Decenoic acid	334-49-6	2084.5	ND	ND	0.92±0.16a	ND	ND	ND	ND
(E)-2-Octenoicacid	1871-67-6	1862.3	ND	ND	1.93±0.51a	ND	ND	ND	ND

2-Hydroxyisobutyric acid	594-61-6	1706.2	ND	ND	1.39±0.18a	ND	ND	ND	ND
Crotonic acid	3724-65-0	1670	ND	ND	ND	ND	ND	ND	0.44±0.05a
2-Heptenoic acid	18999-28-5	1807.7	ND	ND	ND	ND	ND	ND	0.65±0.03a
2-Octenoic acid	1470-50-4	1908	ND	ND	ND	ND	ND	ND	1.53±0.05a
3-Octenoic acid	1577-19-1	1875	ND	ND	ND	ND	ND	ND	0.56±0.01a
Undecylenic acid	112-38-9	2207.6	ND	ND	ND	ND	ND	ND	0.67±0.01a
**Others (14)**									
(2,2-Diethoxyethyl) benzene	6314-97-2	1788.1	ND	ND	2.25±0.43b	4.07±0.56a	1.7±0.12b	ND	ND
2-Methoxy-4-vinylphenol	7786-61-0	2181	2.93±0.23a	ND	ND	2.39±0.05b	ND	ND	0.37±0.07c
(E)-β-Farnesene	18794-84-8	1680	1.02±0.14a	ND	ND	0.71±0.62a	ND	ND	ND
Furfuryl ethyl ether	6270-56-0	1295.8	ND	0.67±0.03a	0.69±0.07a	ND	ND	0.66±0.04a	ND
2-Acetyl pyrrole	1072-83-9	1994.8	ND	ND	ND	ND	ND	ND	1.48±0.16a
2,6-Dimethylpyrazine	108-50-9	1325	ND	ND	ND	ND	ND	ND	0.59±0.13a
Phenol	108-95-2	1989	ND	ND	ND	ND	ND	ND	0.43±0.02a
2-Acetylfuran	1192-62-7	1526.5	ND	ND	ND	ND	ND	ND	3.67±1.09a
1-Ethyl-1H-pyrrole-2-carbaldehyde	2167-14-8	1849.2	ND	ND	ND	ND	ND	ND	0.84±0.04a
2-Propionylfurane	3194-15-8	1595	ND	ND	ND	ND	ND	ND	0.9±0.07a
1-(1,4-dimethylcyclohex-3-en-1-yl) ethan-1-one	43219-68-7	1609.5	ND	ND	ND	ND	ND	ND	0.52±0.11a
Azulene	275-51-4	1743.2	0.58±0.06a	ND	ND	ND	ND	ND	ND
Farnesan	3891-98-3	2036	ND	1.26±0.2a	ND	ND	ND	ND	ND
Styrene	100-42-5	1266.3	ND	ND	ND	ND	ND	6.8±0.02a	ND

Note: "ND" indicates that the compound was not detected. Different letters within the same index denote significant differences among groups at the *p*< 0.05 level. Data are expressed as mean ± standard deviation.
